# Incidence and outcomes of acute high-risk chest pain diseases during pregnancy and puerperium

**DOI:** 10.3389/fcvm.2022.968964

**Published:** 2022-08-11

**Authors:** Shengyong Wu, Xudong Xu, Qian He, Yingyi Qin, Rui Wang, Jun Chen, Chenxin Chen, Cheng Wu, Suxuan Liu

**Affiliations:** ^1^Department of Military Health Statistics, Navy Medical University, Shanghai, China; ^2^Department of Cardiology, Changhai Hospital, Navy Medical University, Shanghai, China; ^3^Department of Gynecology and Obstetrics, Changhai Hospital, Navy Medical University, Shanghai, China

**Keywords:** acute myocardial infarction, aortic dissection, pulmonary embolism, pregnancy, puerperium

## Abstract

**Aim:**

To investigate the incidence and outcomes of acute high-risk chest pain diseases, including acute myocardial infarction (AMI), aortic dissection (AD), and pulmonary embolism (PE) during pregnancy and puerperium.

**Methods:**

The National Inpatient Sample was queried to identify pregnancy-related hospitalizations from January 1, 2008 to December 31, 2017. Temporal trends in the incidence and mortality of AMI, AD and PE were extracted.

**Results:**

Among 41,174,101 hospitalizations, acute high-risk chest pain diseases were diagnosed in 40,285 (0.098%). The incidence increased from 79.92/100,000 in 2008 to 114.79/100,000 in 2017 (P_trend_ < 0.0001). The most frequent was PE (86.5%), followed by AMI (9.6%) and AD (3.3%). The incidence of PE in pregnancy decreased after 2014 and was lower than AMI and AD, while its incidence in puerperium was higher than AMI and AD consistently (P_trend_ < 0.0001). Subgroup analysis showed the incidence of these diseases was higher in black women, lowest-income households, and elderly parturients (P_trend_ < 0.0001). The mortality decreased from 2.24% in 2008 to 2.21% in 2017 (P_trend_ = 0.0240), exhibiting 200-fold higher than patients without these diseases. The following factors were significantly associated with these diseases: aged ≥ 45 years (OR, 4.25; 95%CI, 3.80–4.75), valvular disease (OR, 10.20; 95%CI, 9.73–10.70), and metastatic cancer (OR, 9.75; 95%CI, 7.78–12.22). The trend of elderly parturients increased from 14.94% in 2008 to 17.81% in 2017 (P_trend_ < 0.0001), while no such up-trend was found in valvular disease and metastatic cancer.

**Conclusion:**

The incidence of acute high-risk chest pain diseases, especially PE in puerperium, increased consistently. Although mortality has shown a downward trend, it is still at a high level. We should strengthen monitoring and management of acute high-risk pain diseases in pregnancy and puerperium, especially for black women, lowest-income households, and elderly parturients in the future.

## Highlights

– The incidence of acute high-risk chest pain diseases, especially PE in the puerperium, increased consistently. Among the three diseases, the most frequent disease was PE, almost 10-fold higher than AMI and 26-fold higher than AD.
– The mortality of acute high-risk chest pain diseases slightly decreased from 2.24% in 2008 to 2.21% in 2017, exhibiting 200-fold higher than patients without these diseases. Although mortality has shown a downward trend, it’s still at a high level.
– We highlight that black women, lowest-income households, and advanced maternal age are the risk factors for acute high-risk chest pain diseases during pregnancy and puerperium. Advanced maternal age is a significant risk factor for acute high-risk chest pain diseases, and the number of elderly parturients is still growing. Reducing health care disparities is a huge project for the government and health care system, but at least now, we could increase physician awareness of these high-risk patients.

## Introduction

Acute high-risk chest pain diseases, including acute myocardial infarction (AMI), aortic dissection (AD), and pulmonary embolism (PE), are the major cardiovascular diseases leading to high morbidity and mortality worldwide (1). AMI, AD, and PE have usually been studied and managed as three different diseases. During pregnancy and puerperium, these diseases have a closer relationship involving some common underlying pathophysiological mechanisms (2). Compared with non-pregnant women, pregnant women face a several-fold higher risk of acute high-risk chest pain diseases with potentially devastating outcomes for both the mother and the fetus or baby (3, 4).

Pregnancy puts women into a hypercoagulable state and changes the hemodynamics of the cardiovascular system significantly. In addition, hypertension and diabetes contribute to an increase in the risk of cardiovascular diseases during pregnancy. These risks extend for several months, even into the puerperium period. Acute high-risk chest pain diseases are the leading causes of maternal death in developed countries (5, 6). Early diagnosis and treatment of acute high-risk chest pain diseases are crucial for reducing morbidity and mortality in pregnancy and puerperium.

Much of the previous research focused on just one of the acute high-risk chest pain diseases in pregnancy. Comparing the three diseases and investigating the differences between pregnancy and puerperium are particularly challenging because of the low incidence and heterogeneous clinical presentations. To address these knowledge gaps, we utilized the Nationwide Inpatient Sample (NIS) database to evaluate the incidence, outcomes and risk factors of acute high-risk chest pain diseases during pregnancy and puerperium.

## Materials and methods

### Data source

The NIS database was queried for all patients during pregnancy and puerperium (6 weeks after delivery) between January 1, 2008, to December 31, 2017. The NIS is the largest publicly available all-payer database in the United States which was collected and cleaned by the Healthcare Cost and Utilization Project (HCUP), capturing approximately 20% of inpatient hospitalizations from all community hospitals (7). According to the guidelines of the HCUP, this study does not require Ethical approval because all data from the NIS were de-identified.

### Study population

**FIGURE 1:**
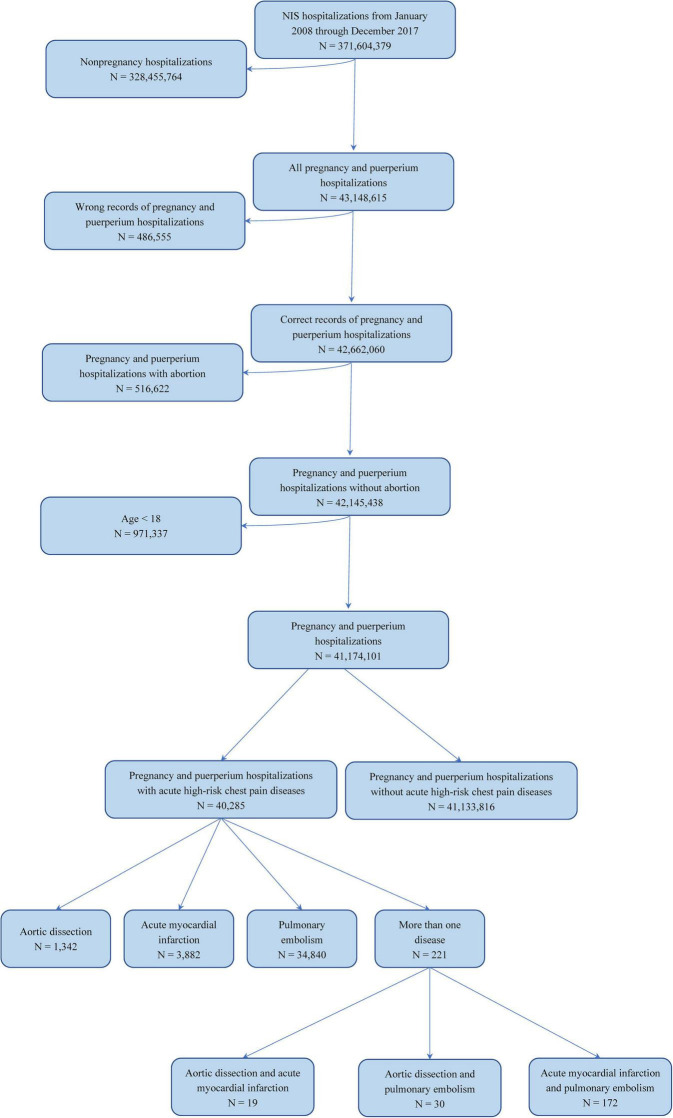
Selection flow diagram of target population. Flow diagram to identify how the study cohort was identified.

Of all 371,604,379 patients contained in the NIS between January 1, 2008, to December 31, 2017, all hospitalizations women aged ≥ 18 years who had diagnoses about pregnancy, labor, or postpartum period were identified using the International Classification of Diseases-9th Revision-Clinical Modification (ICD-9-CM) diagnostic or International Classification of Diseases-10th Revision-Clinical Modification (ICD-10-CM) diagnostic. Acute high-risk chest pain diseases were defined as the diagnosis of hospitalizations containing AD, AMI, or PE, identified by ICD-9-CM or ICD-10-CM (Supplementary Table 1). The selection steps are shown in Figure 1. All demographics and the comorbidities of the hospitalizations and hospital-level characteristics were included in our study.

### Primary and secondary outcomes

The primary outcomes were the temporal trends in the incidence of acute high-risk chest pain diseases during pregnancy and puerperium.

The secondary outcomes included: (1) the temporal trends in the mortality of acute high-risk chest pain diseases during pregnancy and puerperium; (2) risk factors of acute high-risk chest pain diseases during pregnancy and puerperium; (3) the association between acute high-risk chest pain diseases and adverse outcomes, cost, and length of stay (LOS).

### Statistical analysis

The characteristics and demographics were compared between patients with and without acute high-risk chest pain diseases. Using weights provided by NIS, we calculated the overall incidence of acute high-risk chest pain diseases during pregnancy and puerperium. Meanwhile, we analyzed the incidence in subgroups, including status (pregnancy or puerperium), race, household income, and age. The Cochran-Armitage trend test was used to evaluate the significance of trends in these diseases over time. Univariate analysis of the comparisons between two groups was determined by Student’s *t*-test or Mann–Whitney *U* test for normal distributed or non-normal distributed continuous variables, and Pearson Chi-Square test or Mann–Whitney *U* test for unordered categorical variable or ordinal categorical variable.

Using the same covariates, logistic models were constructed to explore the relationship between acute high-risk chest pain diseases and adverse outcomes. Model 1 used the data after missing variables imputed, while model 2 used the original data. To avoid the effect of malignancy on mortality, we built model 3 using data excluding patients with metastatic cancer to explore the relationship between acute high-risk chest pain diseases and death. To evaluate whether the diseases related to the cost and LOS of the hospitalization, we constructed multivariate linear models, adjusting for the same covariate assessment as model 1. Univariate and two multivariate logistic models were constructed to explore the clinical factors associated with acute high-risk chest pain diseases and the three diseases as model 1 and model 2 in all cohort and high incidence subgroups. All tests were two-tailed, and *P* < 0.05 was considered significant unless otherwise specified. Statistical analysis was performed using SAS version 9.4 (SAS Institute Inc.).

### Patient and public involvement

Causing of the nature of this study, there were no patients or the public involved around the design, recruitment, measuring, or writing of the research.

### Missing variables

Most values were missing for < 2% of all hospitalizations, while race and cost were missing for 9.36% and 2.17% (Supplementary Table 2). The missing data of the categorical variables were imputed by the dominant category and the continuous variables by the median.

## Results

### Included population

Among 41,174,101 hospitalizations for pregnancy and puerperium from 2008 to 2017, acute high-risk chest pain diseases were diagnosed in 40,285 patients (0.098%). The most frequent disease was PE (*N* = 34,840, 86.5%), almost 10-fold higher than AMI (*N* = 3,882, 9.6%) and 26-fold higher than AD (*N* = 1,342, 3.3%). A total of 221 patients had more than one disease. A comparison of demographics, hospital characteristics, and outcomes between patients with and without acute high-risk chest pain diseases was presented (Table 1). Patients with acute high-risk chest pain diseases were more likely to be older, black individuals, and had higher proportions of coagulopathy, metastatic cancer, and valvular disease.

**TABLE 1:** Characteristics and outcomes in acute high-risk chest pain diseases during pregnancy and puerperium.

Variables	Acute high-risk chest pain diseases			
	All cohort *N* = 41,174,101	Absent *N* = 41,133,816	Present *N* = 40,285	*P*-value
Age in years at admission	28.32 ± 12.95	28.32 ± 12.95	29.71 ± 13.96	<0.0001
**Age group**				<0.0001
18∼24	11,972,251 (29.08)	11,962,779 (29.08)	9,472 (23.51)	
25∼29	11,996,260 (29.14)	11,985,408 (29.14)	10,851 (26.94)	
30∼34	10,731,812 (26.06)	10,721,402 (26.06)	10,411 (25.84)	
35∼39	5,229,050 (12.70)	5,222,012 (12.70)	7,038 (17.47)	
40∼44	1,160,376 (2.82)	1,158,198 (2.82)	2,178 (5.41)	
≥ 45	84,351 (0.20)	84,016 (0.20)	335 (0.83)	
**Race**				<0.0001
White	23,527,003 (57.14)	23,504,601 (57.14)	22,401 (55.61)	
Black	5,620,816 (13.65)	5,610,510 (13.64)	10,306 (25.58)	
Hispanic	7,893,800 (19.17)	7,888,979 (19.18)	4,821 (11.97)	
Asian or Pacific Islander	2,053,475 (4.99)	2,052,518 (4.99)	957 (2.37)	
Native American	300,573 (0.73)	300,283 (0.73)	290 (0.72)	
Other	1,778,435 (4.32)	1,776,924 (4.32)	1,511 (3.75)	
Elderly parturient women*	6,473,778 (15.72)	6,464,226 (15.72)	9,551 (23.71)	<0.0001
**Median household income percentile**				<0.0001
< P25	11,955,598 (29.04)	11,941,994 (29.03)	13,604 (33.77)	
P25∼P49	10,205,498 (24.79)	10,194,988 (24.78)	10,510 (26.09)	
P50∼P74	10,024,541 (24.35)	10,015,608 (24.35)	8,933 (22.17)	
≥ P75	8,988,464 (21.83)	8,981,226 (21.83)	7,238 (17.97)	
Acquired immune deficiency syndrome	10,952 (0.03)	10,895 (0.03)	56 (0.14)	<0.0001
Alcohol abuse	65,767 (0.16)	65,549 (0.16)	217 (0.54)	<0.0001
Deficiency anemias	3,482,983 (8.46)	3,474,724 (8.45)	8,259 (20.50)	<0.0001
Rheumatoid arthritis/collagen vascular diseases	126,207 (0.31)	125,557 (0.31)	650 (1.61)	<0.0001
Chronic blood loss anemia	4,753,567 (11.55)	4,741,269 (11.53)	12,298 (30.53)	<0.0001
Chronic pulmonary disease	1,724,020 (4.19)	1,719,599 (4.18)	4,421 (10.97)	<0.0001
Coagulopathy	740,143 (1.80)	735,423 (1.79)	4,720 (11.72)	<0.0001
Depression	1,022,485 (2.48)	1,019,691 (2.48)	2,795 (6.94)	<0.0001
Drug abuse	843,984 (2.05)	842,031 (2.05)	1,954 (4.85)	<0.0001
Hypothyroidism	1,117,072 (2.71)	1,115,367 (2.71)	1,705 (4.23)	<0.0001
Liver disease	90,679 (0.22)	90,225 (0.22)	454 (1.13)	<0.0001
Lymphoma	6,908 (0.02)	6,844 (0.02)	64 (0.16)	<0.0001
Metastatic cancer	3,239 (0.01)	3,149 (0.01)	90 (0.22)	<0.0001
Other neurological disorders	361,803 (0.88)	360,351 (0.88)	1,452 (3.60)	<0.0001
Obesity	2,580,366 (6.27)	2,573,861 (6.26)	6,505 (16.15)	<0.0001
Paralysis	22,496 (0.05)	22,226 (0.05)	269 (0.67)	<0.0001
Psychoses	440,467 (1.07)	439,415 (1.07)	1,051 (2.61)	<0.0001
Solid tumor without metastasis	11,373 (0.03)	11,309 (0.03)	64 (0.16)	<0.0001
Peptic ulcer disease excluding bleeding	2,075 (0.01)	2,060 (0.01)	15 (0.04)	<0.0001
Valvular disease	128,712 (0.31)	126,752 (0.31)	1,959 (4.86)	<0.0001
Weight loss	37,874 (0.09)	37,199 (0.09)	675 (1.67)	<0.0001
Gestational hypertension	4,194,875 (10.19)	4,188,029 (10.18)	6,846 (16.99)	<0.0001
Pre-eclampsia/eclampsia	1,858,726 (4.51)	1,855,940 (4.51)	2,786 (6.92)	<0.0001
Gestational diabetes	1,074,398 (2.61)	1,073,111 (2.61)	1,287 (3.20)	<0.0001
Multiple pregnancy	468,147 (1.14)	467,708 (1.14)	439 (1.09)	0.4974
Length of stay days	2.00 (2.00–3.00)	2.00 (2.00–3.00)	4.00 (2.00–6.00)	<0.0001
**Adverse outcomes**				<0.0001
In-hospital death	4,547 (0.01)	3,709 (0.01)	839 (2.08)	<0.0001
Congestive heart failure	48,864 (0.12)	46,601 (0.11)	2,263 (5.62)	<0.0001
Pulmonary circulation disorders^#^	21,083 (0.05)	15,002 (0.04)	6,081 (15.09)	<0.0001
Renal failure	37,043 (0.09)	36,699 (0.09)	344 (0.85)	<0.0001
Fluid and electrolyte disorders	505,867 (1.23)	499,248 (1.21)	6,619 (16.43)	<0.0001

*Elderly parturient women were defined as female during pregnancy or puerperium whose age older than 35-year-old. ^#^Pulmonary circulation disorders were defined as pulmonary embolism and infarction, chronic pulmonary heart disease, and unspecified disease of pulmonary circulation. Causing the weight of observation may not be integer, the count of each group may less or more than the total of all group after rounding off.

### Primary outcomes

**FIGURE 2:**
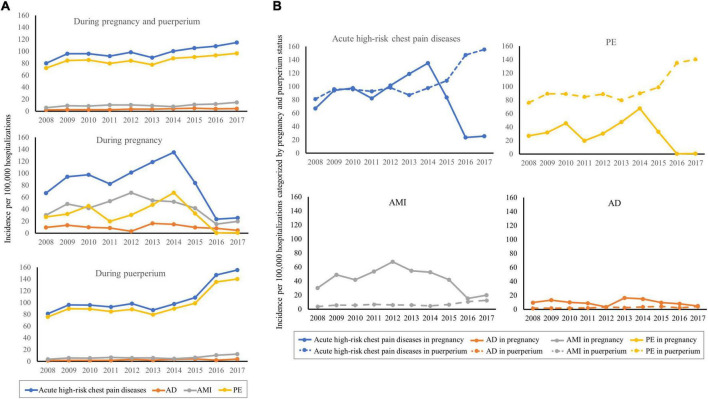
Temporal trends of the incidence in acute high-risk chest pain diseases during pregnancy and puerperium. **(A)** Temporal trends during pregnancy and puerperium, during pregnancy, and during puerperium, respectively (All P_trend_ < 0.0001). **(B)** Temporal trends of the incidence in acute high-risk chest pain diseases, PE, AMI, and AD categorized by pregnancy and puerperium status (All P_trend_ < 0.0001).

As shown in Figure 2A, the incidence of acute high-risk chest pain diseases during pregnancy and puerperium increased from 79.92 per 100,000 hospitalizations in 2008 to 114.79 per 100,000 hospitalizations in 2017 (P_trend_ < 0.0001) (Supplementary Table 3). The incidence of these diseases in pregnancy decreased notably since 2015, while the incidence in puerperium increased consistently (Supplementary Table 4). The incidence of PE in pregnancy decreased after 2014 and was lower than AMI and AD, while its incidence in puerperium was significantly higher than AMI and AD (P_trend_ < 0.0001). As shown in Figure 2B, the incidence of acute high-risk chest pain diseases in puerperium surpassed that in pregnancy since 2015. The incidence of PE in puerperium increased sustainedly and was consistently higher than that in pregnancy. The incidence of AMI and AD in pregnancy was higher than that in the puerperium, respectively.

**FIGURE 3:**
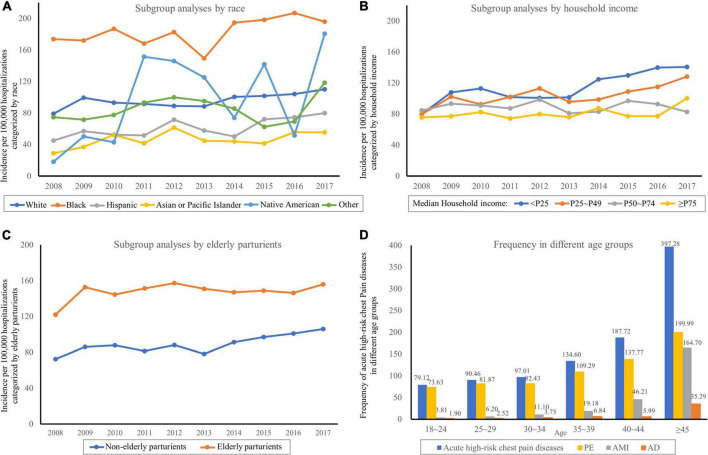
Temporal trends of acute high-risk chest pain diseases in Subgroups. **(A)** Subgroup analyses categorized by race. **(B)** Subgroup analyses categorized by household income. **(C)** Subgroup analyses categorized by elderly parturients. **(D)** The incidence increased with age.

As shown in Figures 3A–C, the subgroup analyses categorized by race, household income, and elderly/non-elderly parturients showed that temporal trends of most subgroups increased. Most of them presented with P_trend_ < 0.0001 (Supplementary Table 4), except for elderly parturient (P_trend_ = 0.0017), Asian or Pacific Islander (P_trend_ = 0.0004), other race (P_trend_ = 0.0213), household income: P50∼P74 (P_trend_ = 0.7509). Black women had the highest incidence, whereas Asian or Pacific Islanders had the lowest incidence. The socio-economic status of patients affected the risk of these diseases. The incidence was highest in the lowest median household income category (0–25th percentile). Elderly parturient women (> 35 years old) were more likely to suffer from these diseases when compared to young women. The incidence increased with age, reaching a peak of 397.28/100,000 hospitalizations at ages over 45 years (Figure 3D).

### Secondary outcomes

**FIGURE 4:**
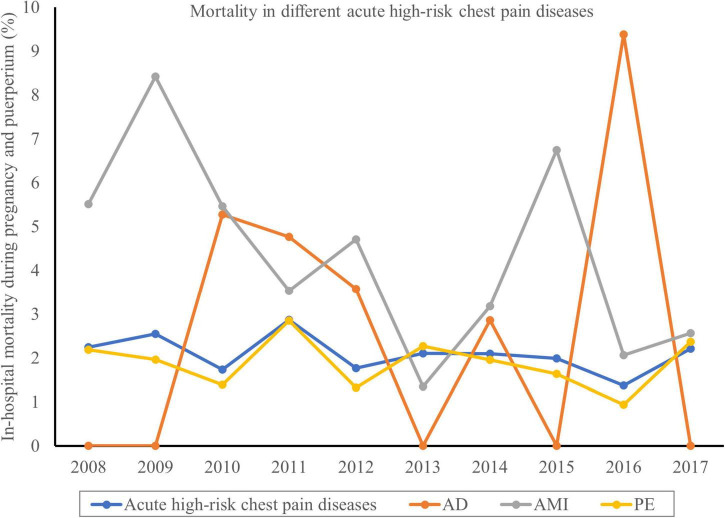
Temporal trends of the mortality in acute high-risk chest pain diseases during pregnancy and puerperium. The in-hospital mortality of acute high-risk chest pain diseases mildly decreased but still remained high (2.24% in 2008 vs. 2.21% in 2017; P_trend_ = 0.0240).

A total of 839 (2.08%) hospitalizations with acute high-risk chest pain diseases died, while the death rate of patients without these diseases was just 0.01% (Table 1). During the study period, the mortality of these diseases mildly decreased but remained high (2.24% in 2008 vs. 2.21% in 2017; P_trend_ = 0.0240) (Supplementary Table 5 and Figure 4). The mortality of AMI decreased from 5.51% in 2008 to 2.56% in 2017 (P_trend_ < 0.0001), while the trends in AD and PE were not statistically significant. Acute high-risk chest pain diseases were associated with an increased cost of $35,789 and an increased LOS of 2.488 days after adjustment confounders (Supplementary Table 6).

After excluding the effect of metastatic cancer on mortality, acute high-risk chest pain diseases were significantly associated with increased in-hospital death (OR, 57.42; 95%CI, 52.43–62.87; Supplementary Table 7). Acute high-risk chest pain diseases were also associated with pulmonary circulation disorders (OR, 255.96; 95%CI, 246.32–265.97; Supplementary Table 8), congestive heart failure (OR, 13.80; 95%CI, 13.09–14.56; Supplementary Table 9), renal failure (OR, 2.80; 95%CI, 2.50–3.14; Supplementary Table 10) and fluid and electrolyte disorders (OR, 8.06; 95%CI, 7.82–8.30; Supplementary Table 11).

The following factors were independently associated with acute high-risk chest pain diseases: over 45 years old (OR, 4.25; 95%CI, 3.80–4.75), valvular disease (OR, 10.20; 95%CI, 9.73–10.70), and metastatic cancer (OR, 9.75; 95%CI, 7.78–12.22) (Table 2). In the further analysis of clinical factors associated with AD, we found that over 45 years old (OR, 7.55; 95%CI, 5.06–11.26) and valvular disease (OR, 86.48; 95%CI, 76.77–97.43) were highly associated with the onset of disease (Supplementary Table 12). The following factors were highly associated with AMI, including over 45 years old (OR, 33.14; 95%CI, 27.16–40.43), valvular disease (OR, 16.82; 95%CI, 15.08–18.76), and metastatic cancer (OR, 11.83; 95%CI, 7.07–19.78) (Supplementary Table 13). The following factors were highly associated with PE, including over 45 years old (OR, 2.34; 95%CI, 2.00–2.73), coagulopathy (OR, 5.23; 95%CI, 5.06–5.41), valvular disease (OR, 6.78; 95%CI, 6.38–7.21) and metastatic cancer (OR, 10.22; 95%CI, 8.05–12.98) (Supplementary Table 14).

**TABLE 2:** Association of clinical factors with acute high-risk chest pain diseases during pregnancy and puerperium.

Variables	Unadjusted model		Multivariate model 1		Multivariate model 2	
	OR (95%CI)	*P*-value	OR (95% CI)	*P*-value	OR (95% CI)	*P*-value
**Age group**						
18∼24–25∼29	1.14 (1.11–1.18)	< 0.0001	1.22 (1.19–1.26)	< 0.0001	1.37 (1.29–1.45)	< 0.0001
18∼24–30∼34	1.23 (1.19–1.26)	< 0.0001	1.38 (1.34–1.42)	< 0.0001	1.76 (1.66–1.87)	< 0.0001
18∼24–35∼39	1.70 (1.65–1.76)	< 0.0001	1.84 (1.78–1.90)	< 0.0001	2.59 (2.43–2.76)	< 0.0001
18∼24–40∼44	2.38 (2.27–2.49)	< 0.0001	2.37 (2.25–2.48)	< 0.0001	3.97 (3.65–4.32)	< 0.0001
18∼24- ≥ 45	5.04 (4.52–5.62)	< 0.0001	4.25 (3.80–4.75)	< 0.0001	11.91 (10.28–13.80)	< 0.0001
**Race**						
White-Black	1.93 (1.88–1.97)	< 0.0001	1.45 (1.42–1.49)	< 0.0001	1.38 (1.32–1.45)	< 0.0001
White-Hispanic	0.64 (0.62–0.66)	< 0.0001	0.62 (0.60–0.64)	< 0.0001	0.61 (0.58–0.65)	< 0.0001
White- Asian or Pacific Islander	0.49 (0.46–0.52)	< 0.0001	0.52 (0.48–0.55)	< 0.0001	0.60 (0.53–0.67)	< 0.0001
White-Native America	1.01 (0.90–1.14)	0.8440	0.93 (0.83–1.04)	0.2070	1.00 (0.80–1.24)	0.9899
White-Other	0.89 (0.85–0.94)	< 0.0001	0.85 (0.81–0.90)	< 0.0001	0.77 (0.69–0.85)	< 0.0001
**Median household income**						
<25–25∼49	0.90 (0.88–0.93)	< 0.0001	1.01 (0.99–1.04)	0.2924	0.93 (0.89–0.98)	0.0051
<25–50∼74	0.78 (0.76–0.80)	< 0.0001	0.88 (0.85–0.90)	< 0.0001	0.74 (0.70–0.78)	< 0.0001
<25- ≥ 75	0.71 (0.69–0.73)	< 0.0001	0.81 (0.78–0.84)	< 0.0001	0.67 (0.63–0.71)	< 0.0001
Gestational hypertension	1.81 (1.76–1.85)	< 0.0001	1.29 (1.25–1.33)	< 0.0001	1.14 (1.08–1.20)	< 0.0001
Pre-eclampsia/eclampsia	1.57 (1.51–1.63)	< 0.0001	0.80 (0.76–0.84)	< 0.0001		
Gestational diabetes	1.23 (1.17–1.30)	< 0.0001	0.81 (0.77–0.86)	< 0.0001	0.73 (0.66–0.82)	< 0.0001
Multiple pregnancy	0.96 (0.87–1.05)	0.3900	0.68 (0.62–0.74)	< 0.0001	0.70 (0.58–0.83)	< 0.0001
Acquired immune deficiency syndrome	5.28 (4.07–6.87)	< 0.0001	1.61 (1.23–2.10)	0.0010	1.89 (1.21–2.93)	0.0047
Alcohol abuse	3.40 (2.97–3.88)	< 0.0001	1.34 (1.17–1.54)	< 0.0001	1.46 (1.18–1.81)	0.0006
Deficiency anemias	2.79 (2.73–2.86)	< 0.0001			1.41 (1.31–1.51)	< 0.0001
Rheumatoid arthritis/collagen vascular diseases	5.36 (4.96–5.80)	< 0.0001	2.35 (2.17–2.55)	< 0.0001	2.04 (1.75–2.37)	< 0.0001
Chronic blood loss anemia	3.37 (3.30–3.45)	< 0.0001	2.49 (2.44–2.55)	< 0.0001	1.54 (1.44–1.64)	< 0.0001
Chronic pulmonary disease	2.83 (2.74–2.92)	< 0.0001	1.73 (1.68–1.79)	< 0.0001	1.37 (1.28–1.46)	< 0.0001
Coagulopathy	7.29 (7.07–7.52)	< 0.0001	4.99 (4.83–5.15)	< 0.0001	4.45 (4.19–4.73)	< 0.0001
Depression	2.93 (2.82–3.05)	< 0.0001	1.77 (1.70–1.84)	< 0.0001	1.81 (1.68–1.95)	< 0.0001
Drug abuse	2.44 (2.33–2.55)	< 0.0001	1.39 (1.32–1.46)	< 0.0001	2.12 (1.96–2.29)	< 0.0001
Hypothyroidism	1.59 (1.51–1.66)	< 0.0001	1.22 (1.16–1.28)	< 0.0001		
Liver disease	5.19 (4.73–5.69)	< 0.0001	2.27 (2.07–2.50)	< 0.0001	2.22 (1.89–2.61)	< 0.0001
Lymphoma	9.58 (7.49–12.25)	< 0.0001	4.07 (3.13–5.28)	< 0.0001		
Metastatic cancer	29.16 (23.63–35.97)	< 0.0001	9.75 (7.78–12.22)	< 0.0001	17.46 (12.97–23.51)	< 0.0001
Other neurological disorders	4.23 (4.02–4.46)	< 0.0001	2.05 (1.94–2.16)	< 0.0001	2.54 (2.32–2.78)	< 0.0001
Obesity	2.89 (2.81–2.96)	< 0.0001	1.98 (1.92–2.04)	< 0.0001	2.46 (2.34–2.58)	< 0.0001
Paralysis	12.44 (11.03–14.04)	< 0.0001	3.92 (3.44–4.46)	< 0.0001	3.44 (2.75–4.30)	< 0.0001
Psychoses	2.48 (2.33–2.64)	< 0.0001	1.28 (1.20–1.36)	< 0.0001	1.21 (1.07–1.35)	0.0016
Solid tumor without metastasis	5.81 (4.55–7.43)	< 0.0001	3.28 (2.55–4.22)	< 0.0001	4.89 (3.38–7.07)	< 0.0001
Peptic ulcer disease excluding bleeding	7.46 (4.49–12.39)	< 0.0001	2.09 (1.23–3.54)	0.0061		
Valvular disease	16.54 (15.80–17.31)	< 0.0001	10.20 (9.73–10.70)	< 0.0001	21.26 (19.91–22.69)	< 0.0001
Weight loss	18.82 (17.43–20.33)	< 0.0001	6.65 (6.12–7.22)	< 0.0001	8.39 (7.38–9.53)	< 0.0001

The trends of most clinical factors increased from 2008 to 2017 (all P_trend_ < 0.0001, Supplementary Table 15), except valvular disease, lymphoma, and metastatic cancer. The trend of elderly parturients increased from 14.94% in 2008 to 17.81% in 2017 (P_trend_ < 0.0001).

## Discussion

In this nationwide observational analysis of 41,174,101 hospitalizations for pregnancy and puerperium from 2008 to 2017, acute high-risk chest pain diseases were diagnosed in 40,285 patients (0.098%). The incidence increased from 79.92/100,000 in 2008 to 114.79/100,000 in 2017. The incidence of acute high-risk chest pain diseases in puerperium consistently increased and even surpassed the incidence in pregnancy since 2015. Among the three diseases, the most frequent disease was PE, almost 10-fold higher than AMI and 26-fold higher than AD. The incidence of PE in puerperium increased consistently and was significantly higher than that in pregnancy. The high portion of PE among the three diseases and its increased incidence in puerperium lead to the overall upward trend of acute high-risk chest pain diseases in puerperium. The mortality of acute high-risk chest pain diseases slightly decreased from 2.24% in 2008 to 2.21% in 2017, exhibiting 200-fold higher than patients without these diseases. The following factors were significantly associated with acute high-risk chest pain diseases: aged ≥ 45 years (OR, 4.25; 95%CI, 3.80–4.75), valvular disease (OR, 10.20; 95%CI, 9.73–10.70), and metastatic cancer (OR, 9.75; 95%CI, 7.78–12.22). The trend of elderly parturients increased from 14.94% in 2008 to 17.81% in 2017 (P_trend_ < 0.0001), while no such up-trend was found in valvular disease and metastatic cancer.

Pregnancy is a hypercoagulable state characterized by increased prothrombotic factors. In addition, dilated veins, decreased or obstructed venous flow by the enlarging uterus lead to deep vein thrombosis and pregnancy-related PE. PE is the first leading cause of maternal death in the United Kingdom and the sixth in the United States (8, 9). In our data, the incidence of PE was 0.0846% and was significantly higher than AD and AMI. The incidence we presented was mildly higher than the previously reported incidence of 0.03% (5). Elgendy et al. (10) reported that the incidence of PE remained unchanged from 2007 to 2015. We found that the incidence of PE in puerperium increased consistently from 2008 to 2017 and was significantly higher than that in pregnancy. This can be due to multiple reasons. First, not only the PE, but we also observed a decrease in AMI and AD during pregnancy. We believe this results from adequate attention to these diseases during pregnancy over the past 20 years. Second, vascular trauma, assistive devices, and cesarean section heighten postpartum thrombotic risk during delivery (11). The risk of venous thromboembolism increases in the immediate postpartum period and returns to the non-pregnant level until the sixth week postpartum (5). In a previous population-based study from 1966 to 1995, the postpartum period was also found to be the highest risk period for PE (3). We confirmed the high risk of PE in puerperium and demonstrated its continuous upward trend from 2008 to 2017. Management of PE in puerperium may not be given sufficient attention after discharge and should be strengthened in the future.

Mechanisms of AMI in pregnancy include plaque rupture or erosion, coronary dissection, and thrombus (12, 13). In our data, the incidence of AMI during pregnancy and puerperium increased from 5.9/100,000 in 2008 to 14.8/100,000 in 2017. Similar findings reported that the incidence of AMI in pregnancy and puerperium increased from 7.1/100,000 in 2002 to 9.5/100,000 in 2014 (P_trend_ < 0.001) (14). Accordingly, we concluded that the incidence of AMI increased from 2002 to 2017. Moreover, we could not ignore that some women with suspected AMI during pregnancy did not undergo coronary angiography for radiation risks to the mother and fetus. In addition, detailed angiographic findings or intracoronary imaging results were not available for patients who underwent invasive management due to the limitation of ICD-9/10 codes from the NIS data set. We could not identify the mechanisms of AMI from the available data. The true incidence and mechanisms of AMI during pregnancy and puerperium warrants further exploration.

In our study, the incidence of AD in pregnancy and puerperium was 0.003%, which was the lowest among the three diseases. The rate of AD in pregnancy was reported at 0.0004% between 1998 and 2008, representing 0.1% of all cases of AD (15). Population-based studies demonstrated a similarly rare AD occurrence in pregnancy (16, 17). Although rare, the incidence of AD has increased in the past 20 years. We believe that the period of pregnancy and puerperium are portended independent risk time for AD. During pregnancy, high cardiac output, hormonal changes, gestational hypertension, hereditary connective tissue diseases, coarctation of the aorta, and previous aortic surgery increase AD susceptibility (18–20). Sometimes, AD symptoms may mimic AMI and PE, resulting in inadvertent use of antiplatelet and anticoagulant therapy and devastating outcomes. Thus, we must always maintain a strong suspicion of high-risk women prone to AD during pregnancy and puerperium.

The increasing incidences of acute high-risk chest pain diseases indicate we must identify the risk factors behind the rise, including modifiable and unmodifiable factors. Previous population-based studies reported that maternal age, hypertension, and diabetes were independent risk factors associated with AMI during pregnancy (4, 21). We observed a consistently higher incidence of acute high-risk chest pain diseases in the elderly (> 35 years old) than non-elderly parturient women. The incidence increased with age, reaching a peak of 397.28/100,000 hospitalizations at ages over 45 years. In the multivariable analysis, age over 45 was closely related to acute high-risk chest pain diseases (OR, 4.25; 95%CI, 3.80–4.75). Compared with AD (OR, 7.55; 95%CI, 5.06–11.26) and PE (OR, 2.34; 95%CI, 2.00–2.73), age over 45 years old was significantly associated with AMI (OR, 33.14; 95%CI, 27.16–40.43). A previous study reported that advanced maternal age was closely related to AMI, with up to 30-fold odds in pregnant women aged ≥ 40 than women < 20 years (4, 22). These data are in agreement with our results. We also found that the trend of elderly parturients increased from 14.94% in 2008 to 17.81% in 2017 (P_trend_ < 0.0001). Our findings highlight that advanced maternal age is a significant risk factor for acute high-risk chest pain diseases, especially AMI, and the number of elderly parturients is still increasing. The upward trend in the number of elderly parturients is of concern. Maternal health policies should focus on advanced maternal age pregnancies in the future.

Besides age, race is another unmodifiable factor for acute high-risk chest pain diseases. Previous studies reported that the black race was independently associated with AMI during pregnancy and puerperium (12, 14). In pregnant women with PE, the black race was contributed to a higher proportion of deaths than white women from 2007 to 2016 (23). In our study, black women were significantly associated with acute high-risk chest pain diseases (OR, 1.44; 95% CI, 1.40–1.48). In the multivariable analysis of each disease, the black race was highly associated with AMI (OR, 1.65; 95%CI, 1.52–1.79) and PE (OR, 1.48; 95%CI, 1.44–1.52). Compared with white women, black women had a low risk of AD (OR, 0.53; 95%CI, 0.45–0.63), while native Americans were highly associated with AD (OR, 1.56; 95%CI, 0.99–2.47). However, due to the rare incidence of AD in pregnancy, the association between race and AD warrants further investigation. The socio-economic status of patients also affected the risk of acute high-risk chest pain diseases. The incidence was highest in the lowest median household income category (0 to 25th percentile). Our study may inform interventions to promote health equity for patients with the lowest median household income. Reducing health care disparities is a huge project for the government and health care system, but at least now, we could increase physician awareness of these high-risk patients.

In multivariable analysis, valvular disease (OR, 10.20; 95%CI, 9.73–10.70) and metastatic cancer (OR, 9.75; 95%CI, 7.78–12.22) were independently associated with acute high-risk chest pain diseases. The valvular disease can be rheumatic, congenital, or degenerative during pregnancy (5). In particular, valvular disease was significantly associated with AD (OR, 86.48; 95%CI, 76.77–97.43). Valvular disease is a common cause inducing AD. Pregnant woman with valvular diseases, especially aortic stenosis and regurgitation, is at increased risk for aortic aneurysms and AD (24). Malignancy is a primary reason leading to coagulation system activation (25). In turn, coagulation activation and platelet aggregation might facilitate metastasis. Our study found that metastatic cancer was significantly related to AMI (OR, 11.83; 95%CI, 7.07–19.78) and PE (OR, 10.22; 95%CI, 8.05–12.98), and there was no association between metastatic cancer and AD.

Moreover, we found that gestational hypertension was positively associated with acute high-risk chest pain diseases (OR, 1.29; 95%CI, 1.25–1.33) while gestational diabetes was negatively associated with acute high-risk chest pain diseases (OR, 0.81; 95%CI, 0.77–0.86). The incidence of driving risk factors is different in different races and regions, which makes the occurrence of diseases in various manners. Asians and Pacific Islanders comprise 5% of the total population in the United States and have a high incidence of gestational diabetes. However, we found that Asian and Pacific Islanders had the lowest incidence of acute high-risk chest pain diseases, whereas black women had the highest incidence. Our study seems inconsistent with previous findings that hypertension and diabetes are strong risk factors for cardiovascular diseases. We speculate that patients with gestational hypertension or diabetes might attract more attention due to the high risk leading to cardiovascular complications and thus improve outcomes in these patients. Taken together, the interaction of risk factors and concomitant diseases can lead to the development of acute high-risk chest pain diseases. Management of risk factors and concomitant diseases may reduce the incidence of acute high-risk chest pain diseases in pregnant or puerperal women.

In brief, we highlight that black women, lowest-income households, advanced maternal age, valvular disease, and metastatic cancer are the risk factors of acute high-risk chest pain diseases during pregnancy and puerperium. The trend of elderly parturients increased from 2008 to 2017, while no such up-trend was found in valvular disease and metastatic cancer. A recent survey has revealed that cardiovascular disease is the leading cause of pregnancy-related deaths in the United States, accounting for 15.5% of all pregnancy-related deaths from 2014 to 2017 (9). In our study, acute high-risk chest pain diseases were significantly associated with increased in-hospital death after excluding the effect of metastatic cancer. Although the mortality has shown a downward trend (2.24% in 2008 vs. 2.21% in 2017), it is still at a high level with a 200-fold higher risk. Among these diseases, the mortality of AMI decreased from 5.51% in 2008 to 2.56% in 2017. No such trend was found in AD and PE patients. Thus, more efforts should be made to reduce mortality among these populations. Sufficient attention should be paid to patients presenting chest pain during pregnancy and puerperium, especially for black women, lowest-income households, and elderly parturients.

## Limitations

Despite its strengths, there are several limitations to the current analysis. First, limited by the data elements of the NIS database, some vital information such as medication administration, procedural details, and genetic testing are not collected, which may cause bias in outcome evaluation. Second, it should be noted that approximately one-third of pregnancy-related deaths occurred outside a medical facility (26). These patients who died outside the hospital would not be reported in the NIS and represent missing data. Third, the NIS database is representative of the American population. The incidence of driving risk factors is different in different races and regions. Our results may not be generalized to the populations outside the United States. Fourth, due to the low incidence of AD, there were only 1,342 patients queried from the database, which may lead to dramatic fluctuations in mortality and biased estimates of overall trends. Fifth, the diagnoses of each disease relying on ICD codes may have coding errors and cannot be confirmed from standard criteria. Sixth, there is a coding shift from ICD-9 to ICD-10 in 2015. Considering this, we queried the variables to be included in the initial trial design process of the study with ICD-9 and ICD-10 respectively, achieving one-to-one correspondence and all relevant codes included as possible. Despite these limitations, our study offers essential information on acute high-risk chest pain diseases using a nationally representative sample of women during pregnancy and puerperium. This database reflects the current real-world practice and avoids the potential biases originating from specialized centers.

## Conclusion

In conclusion, we found that the incidence of acute high-risk chest pain diseases in puerperium consistently increased during the study period and even surpassed the incidence in pregnancy since 2015. The most frequent disease was PE, and the incidence of PE in puerperium increased consistently. Although the mortality of acute high-risk chest pain diseases has shown a downward trend, it is still at a high level with a 200-fold higher risk. Advanced maternal age is a significant risk factor for acute high-risk chest pain diseases, and the number of elderly parturients is still growing. We should strengthen monitoring and management of acute high-risk pain diseases in pregnancy and puerperium, especially for black women, lowest-income households, and elderly parturients in the future.

## Data availability statement

The original contributions presented in this study are included in the article/Supplementary material, further inquiries can be directed to the corresponding author/s.

## Ethics statement

Ethical review and approval was not required for the study on human participants in accordance with the local legislation and institutional requirements. Written informed consent for participation was not required for this study in accordance with the national legislation and the institutional requirements.

## Author contributions

SW, XX, QH, YQ, RW, JC, and CC had full access to the data and verified the data. SL and CW were responsible for the decision to submit the manuscript and contributed to the concept, design, and supervision. SW and XX drafted the manuscript. QH, YQ, and RW contributed to the statistical analysis. All authors contributed to critical revision of the manuscript for important intellectual content.
