# Supplementary material for: Incidence and outcomes of acute high-risk chest pain diseases during pregnancy and puerperium
Source: Front Cardiovasc Med. 2022 Aug 11;9:968964. doi: 10.3389/fcvm.2022.968964 (PMC9403474; doi:10.3389/fcvm.2022.968964)
Supplement: Supplementary file 1 [file Data_Sheet_1.PDF]

Supplementary Tables

Supplementary Table 1. ICD Codes of the Acute High-risk Chest Pain Diseases, Abortion, Valvular Diseases, Pregnancy and Puerperium

Supplementary Table 2. Missing Data Situations and Filling Strategies

Supplementary Table 3. Temporal Trends of the Incidence of Acute High-risk Chest Pain Diseases per 100,000 Patients During Pregnancy and Puerperium

Supplementary Table 4. Temporal Trends of the Incidence of Subgroups Which Categorized by Age, Status, Race, Household income, Insurance type, and Region, per 100,000 Patients

Supplementary Table 5. Temporal Trends of the In-Hospital Mortality of Acute High-risk Chest Pain Diseases During Pregnancy and Puerperium

Supplementary Table 6. Association of Hospitalization Cost and LOS with Acute High-risk Chest Pain Diseases During Pregnancy and Puerperium

Supplementary Table 7. Association of In-hospital Death with Acute High-risk Chest Pain Diseases and Clinical Factors

Supplementary Table 8. Association of Pulmonary Circulation Disorders with Acute High-risk Chest Pain Diseases and Clinical Factors

Supplementary Table 9. Association of Congestive Heart Failure with Acute High-risk Chest Pain Diseases and Clinical Factors

Supplementary Table 10. Association of Renal Failure with Acute High-risk Chest Pain Diseases and Clinical Factors

Supplementary Table 11. Association of Fluid and Electrolyte Disorders with Acute High-risk Chest Pain Diseases and Clinical Factors

Supplementary Table 12. Association of Clinical Factors with Aortic Dissection During Pregnancy and Puerperium

Supplementary Table 13. Association of Clinical Factors with Acute Myocardial Infarction During Pregnancy and Puerperium

Supplementary Table 14. Association of Clinical Factors with Pulmonary Embolism During Pregnancy and Puerperium

Supplementary Table 15. Temporal Trends of Factors Associated with Acute High-risk Chest Pain Diseases During Pregnancy and Puerperium

Supplementary Table 1. ICD Codes of the Acute High-risk Chest Pain Diseases, Abortion, Valvular Diseases, Pregnancy and Puerperium

Disease	ICD-9 disease classification	ICD-9 Code	ICD-10 disease classification	ICD-10 Code
Aortic dissection	Dissection of aorta [any part]	441.0	Dissection of aorta [any part]	I71.0
Aortic dissection	Thoracic aortic aneurysm, ruptured	441.1	Thoracic aortic aneurysm, ruptured	I71.1
Aortic dissection	Thoracic aortic aneurysm, without mention of rupture	441.2	Thoracic aortic aneurysm, without mention of rupture	I71.2
Aortic dissection	Abdominal aortic aneurysm, ruptured	441.3	Abdominal aortic aneurysm, ruptured	I71.3
Aortic dissection	Abdominal aortic aneurysm, without mention of rupture	441.4	Abdominal aortic aneurysm, without mention of rupture	I71.4
Aortic dissection	Thoracoabdominal aortic aneurysm, ruptured	441.5	Thoracoabdominal aortic aneurysm, ruptured	I71.5
Aortic dissection	Thoracoabdominal aortic aneurysm, without mention of rupture	441.6	Thoracoabdominal aortic aneurysm, without mention of rupture	I71.6
Aortic dissection	Aortic aneurysm of unspecified site, ruptured	441.7	Aortic aneurysm of unspecified site, ruptured	I71.7
Aortic dissection	Aortic aneurysm of unspecified site, without mention of rupture	441.9	Aortic aneurysm of unspecified site, without mention of rupture	I71.9
Acute myocardial infarction	Acute myocardial infarction: Of anterolateral wall	410.0	Acute transmural myocardial infarction of anterior wall	I21.0
Acute myocardial infarction	Acute myocardial infarction: Of other anterior wall	410.1	Acute transmural myocardial infarction of inferior wall	I21.1
Acute myocardial infarction	Acute myocardial infarction: Of inferolateral wall	410.2	Acute transmural myocardial infarction of other sites	I21.2
Acute myocardial infarction	Acute myocardial infarction: Of infer posterior wall	410.3	Acute transmural myocardial infarction of unspecified site	I21.3
Acute myocardial infarction	Acute myocardial infarction: Of other inferior wall	410.4	Acute subendocardial myocardial infarction	I21.4
Acute myocardial infarction	Acute myocardial infarction: Of other lateral wall	410.5	Acute myocardial infarction, unspecified	I21.9
Acute myocardial infarction	Acute myocardial infarction: True posterior wall infarction	410.6	Subsequent myocardial infarction of anterior wall	I22.0
Acute myocardial infarction	Acute myocardial infarction: Subendocardial infarction	410.7	Subsequent myocardial infarction of inferior wall	I22.1
Acute myocardial infarction	Acute myocardial infarction: Of other specified sites	410.8	Subsequent myocardial infarction of other sites	I22.8
Acute myocardial infarction	Acute myocardial infarction: Unspecified site	410.9	Subsequent myocardial infarction of unspecified site	I22.9
Pulmonary embolism	Acute cor pulmonale	415.0	Pulmonary embolism with mention of acute cor pulmonale	I26.0
Pulmonary embolism	Pulmonary embolism and infarction	415.1	Obstetric air embolism	O88.0
Pulmonary embolism	Obstetrical air embolism	673.0	Amniotic fluid embolism	O88.1
Pulmonary embolism	Amniotic fluid embolism	673.1	Obstetric blood-clot embolism	O88.2
Pulmonary embolism	Obstetrical blood-clot embolism	673.2	Obstetric pyemic and septic embolism	O88.3

Disease	ICD-9 disease classification	ICD-9 Code	ICD-10 disease classification	ICD-10 Code
Pulmonary embolism	Obstetrical pyemic and septic embolism	673.3	Other obstetric embolism	O88.8
Pulmonary embolism	Other pulmonary embolism	673.8		
Valvular diseases	Mitral stenosis	394.0	Rheumatic mitral stenosis	I05.0
Valvular diseases	Rheumatic mitral insufficiency	394.1	Rheumatic mitral insufficiency	I05.1
Valvular diseases	Mitral stenosis with insufficiency	394.2	Rheumatic mitral stenosis with insufficiency	I05.2
Valvular diseases	Other and unspecified mitral valve diseases	394.9	Other rheumatic mitral valve diseases	I05.8
Valvular diseases	Rheumatic aortic stenosis	395.0	Rheumatic mitral valve disease, unspecified	I05.9
Valvular diseases	Rheumatic aortic insufficiency	395.1	Rheumatic aortic stenosis	I06.0
Valvular diseases	Rheumatic aortic stenosis with insufficiency	395.2	Rheumatic aortic insufficiency	I06.1
Valvular diseases	Other and unspecified rheumatic aortic diseases	395.9	Rheumatic aortic stenosis with insufficiency	I06.2
Valvular diseases	Mitral valve stenosis and aortic valve stenosis	396.0	Rheumatic aortic stenosis with insufficiency	I06.8
Valvular diseases	Mitral valve stenosis and aortic valve insufficiency	396.1	Rheumatic aortic valve disease, unspecified	I06.9
Valvular diseases	Mitral valve insufficiency and aortic valve stenosis	396.2	Rheumatic disorders of both mitral and aortic valves	I08.0
Valvular diseases	Mitral valve insufficiency and aortic valve insufficiency	396.3	Rheumatic disorders of both mitral and tricuspid valves	I08.1
Valvular diseases	Multiple involvement of mitral and aortic valves	396.8	Rheumatic disorders of both aortic and tricuspid valves	I08.2
Valvular diseases	Mitral and aortic valve diseases, unspecified	396.9	Combined rheumatic disorders of mitral, aortic and tricuspid valves	I08.3
Valvular diseases	Rheumatic diseases of pulmonary valve	397.1	Other rheumatic multiple valve diseases	I08.8
Valvular diseases	Rheumatic diseases of endocardium, valve unspecified	397.9	Rheumatic multiple valve disease, unspecified	I08.9
Valvular diseases	Mitral valve disorders	424.0	Rheumatic diseases of endocardium, valve unspecified	I09.1
Valvular diseases	Aortic valve disorders	424.1	Other specified rheumatic heart diseases	I09.89
Valvular diseases	Tricuspid valve disorders, specified as nonrheumatic	424.2	Nonrheumatic mitral (valve) insufficiency	I34.0
Valvular diseases	Pulmonary valve disorders	424.3	Nonrheumatic mitral (valve) prolapse	I34.1
Valvular diseases	Endocarditis valve unspecified	424.9	Nonrheumatic mitral (valve) stenosis	I34.2
Valvular diseases	Congenital stenosis of aortic valve	746.3	Other nonrheumatic mitral valve disorders	I34.8
Valvular diseases	Congenital insufficiency of aortic valve	746.4	Nonrheumatic mitral valve disorder, unspecified	I34.9

Disease	ICD-9 disease classification	ICD-9 Code	ICD-10 disease classification	ICD-10 Code
Valvular diseases	Congenital mitral stenosis	746.5	Nonrheumatic aortic (valve) stenosis	I35.0
Valvular diseases	Congenital mitral insufficiency	746.6	Nonrheumatic aortic (valve) insufficiency	I35.1
Valvular diseases	Organ or tissue replaced by transplant: Heart valve	V42.2	Nonrheumatic aortic (valve) stenosis with insufficiency	I35.2
Valvular diseases	Organ or tissue replaced by other means: Heart valve	V43.3	Other nonrheumatic aortic valve disorders	I35.8
Valvular diseases	Syphilitic endocarditis of valve, unspecified	093.20	Nonrheumatic aortic valve disorder, unspecified	I35.9
Valvular diseases	Syphilitic endocarditis of mitral valve	093.21	Nonrheumatic tricuspid (valve) stenosis	I36.0
Valvular diseases	Syphilitic endocarditis of aortic valve	093.22	Nonrheumatic tricuspid (valve) insufficiency	I36.1
Valvular diseases	Syphilitic endocarditis of tricuspid valve	093.23	Nonrheumatic tricuspid (valve) stenosis with insufficiency	I36.2
Valvular diseases	Syphilitic endocarditis of pulmonary valve	093.24	Other nonrheumatic tricuspid valve disorders	I36.8
Valvular diseases			Nonrheumatic tricuspid valve disorder, unspecified	I36.9
Valvular diseases			Nonrheumatic pulmonary valve stenosis	I37.0
Valvular diseases			Nonrheumatic pulmonary valve insufficiency	I37.1
Valvular diseases			Nonrheumatic pulmonary valve stenosis with insufficiency	I37.2
Valvular diseases			Other nonrheumatic pulmonary valve disorders	I37.8
Valvular diseases			Nonrheumatic pulmonary valve disorder, unspecified	I37.9
Valvular diseases			Endocarditis, valve unspecified	I38
Valvular diseases			Congenital stenosis of aortic valve	Q23.0
Valvular diseases			Congenital insufficiency of aortic valve	Q23.1
Valvular diseases			Congenital mitral stenosis	Q23.2
Valvular diseases			Congenital mitral insufficiency	Q23.3
Valvular diseases			Presence of prosthetic heart valve	Z95.2
Valvular diseases			Presence of xenogenic heart valve	Z95.3
Valvular diseases			Syphilitic endocarditis	A52.03

Supplementary Table 1. Continued ICD Codes of the Acute High-risk Chest Pain Diseases, Abortion, Pregnancy and Puerperium

Status	ICD-9 disease classification	ICD-9 Code	ICD-10 disease classification	ICD-10 Code
Abortion	Hydatidiform mole	630	Ectopic pregnancy	O00
Abortion	Other abnormal product of conception	631	Hydatidiform mole	O01
Abortion	Missed abortion	632	Other abnormal products of conception	O02
Abortion	Ectopic pregnancy	633	Spontaneous abortion	O03
Abortion	Spontaneous abortion	634	Medical abortion	O04
Abortion	Legally induced abortion	635	Other abortion	O05
Abortion	Illegally induced abortion	636	Unspecified abortion	O06
Abortion	Unspecified abortion	637	Failed attempted abortion	O07
Abortion	Failed attempted abortion	638	Complications following abortion and ectopic and molar pregnancy	O08
Abortion	Complications following abortion and ectopic and molar pregnancies	639		
Pregnancy or Puerperium	Hemorrhage in early pregnancy	640	Pre-existing hypertension complicating pregnancy, childbirth and the puerperium	O10
Pregnancy or Puerperium	Antepartum hemorrhage, abruptio placentae, and placenta previa	641	Pre-existing hypertensive disorder with superimposed proteinuria	O11
Pregnancy or Puerperium	Hypertension complicating pregnancy, childbirth, and the puerperium	642	Gestational [pregnancy-induced] oedema and proteinuria without hypertension	O12
Pregnancy or Puerperium	Excessive vomiting in pregnancy	643	Gestational [pregnancy-induced] hypertension without significant proteinuria	O13
Pregnancy or Puerperium	Early or threatened labor	644	Gestational [pregnancy-induced] hypertension with significant proteinuria	O14
Pregnancy or Puerperium	Late pregnancy	645	Eclampsia	O15
Pregnancy or Puerperium	Other complications of pregnancy, not elsewhere classified	646	Unspecified maternal hypertension	O16
Pregnancy or Puerperium	Infectious and parasitic conditions in the mother classifiable elsewhere, but complicating pregnancy, childbirth, or the puerperium	647	Haemorrhage in early pregnancy	O20
Pregnancy or Puerperium	Other current conditions in the mother classifiable elsewhere, but complicating pregnancy, childbirth, or the puerperium	648	Excessive vomiting in pregnancy	O21

Status	ICD-9 disease classification	ICD-9 Code	ICD-10 disease classification	ICD-10 Code
Pregnancy or Puerperium	Other conditions or status of the mother complicating pregnancy, childbirth, or the puerperium	649	Venous complications in pregnancy	O22
Pregnancy or Puerperium	Normal delivery	650	Infections of genitourinary tract in pregnancy	O23
Pregnancy or Puerperium	Multiple gestation	651	Diabetes mellitus in pregnancy	O24
Pregnancy or Puerperium	Malposition and malpresentation of fetus	652	Malnutrition in pregnancy	O25
Pregnancy or Puerperium	Disproportion	653	Maternal care for other conditions predominantly related to pregnancy	O26
Pregnancy or Puerperium	Abnormality of organs and soft tissues of pelvis	654	Abnormal findings on antenatal screening of mother	O28
Pregnancy or Puerperium	Known or suspected fetal abnormality affecting management of mother	655	Complications of anaesthesia during pregnancy	O29
Pregnancy or Puerperium	Other known or suspected fetal and placental problems affecting management of mother	656	Multiple gestation	O30
Pregnancy or Puerperium	Polyhydramnios	657	Complications specific to multiple gestation	O31
Pregnancy or Puerperium	Other problems associated with amniotic cavity and membranes	658	Maternal care for known or suspected malpresentation of fetus	O32
Pregnancy or Puerperium	Other indications for care or intervention related to labor and delivery, not elsewhere classified	659	Maternal care for known or suspected disproportion	O33
Pregnancy or Puerperium	Health supervision of infant or child	V20	Maternal care for known or suspected abnormality of pelvic organs	O34
Pregnancy or Puerperium	Constitutional states in development	V21	Maternal care for known or suspected fetal abnormality and damage	O35
Pregnancy or Puerperium	Normal pregnancy	V22	Maternal care for other known or suspected fetal problems	O36
Pregnancy or Puerperium	Supervision of high-risk pregnancy	V23	Polyhydramnios	O40
Pregnancy or Puerperium	Postpartum care and examination	V24	Other disorders of amniotic fluid and membranes	O41
Pregnancy or Puerperium	Encounter for contraceptive management	V25	Premature rupture of membranes	O42
Pregnancy or Puerperium	Procreative management	V26	Placental disorders	O43
Pregnancy or Puerperium	Outcome of delivery	V27	Placenta praevia	O44
Pregnancy or Puerperium	Encounter for antenatal screening of mother	V28	Premature separation of placenta [abruptio placentae]	O45
Pregnancy or Puerperium	Observation and evaluation of newborns for suspected condition not found	V29	Antepartum haemorrhage, not elsewhere classified	O46
Pregnancy or Puerperium	Single liveborn	V30	False labour	O47

Status	ICD-9 disease classification	ICD-9 Code	ICD-10 disease classification	ICD-10 Code
Pregnancy or Puerperium	Twin, mate liveborn	V31	Prolonged pregnancy	O48
Pregnancy or Puerperium	Twin, mate stillborn	V32	Preterm labour and delivery	O60
Pregnancy or Puerperium	Twin, unspecified	V33	Failed induction of labour	O61
Pregnancy or Puerperium	Other multiple, mates all liveborn	V34	Abnormalities of forces of labour	O62
Pregnancy or Puerperium	Other multiple, mates all stillborn	V35	Long labour	O63
Pregnancy or Puerperium	Other multiple, mates live- and stillborn	V36	Obstructed labour due to malposition and malpresentation of fetus	O64
Pregnancy or Puerperium	Other multiple, unspecified	V37	Obstructed labour due to maternal pelvic abnormality	O65
Pregnancy or Puerperium	Unspecified	V39	Other obstructed labour	O66
Pregnancy or Puerperium			Labour and delivery complicated by intrapartum haemorrhage, not elsewhere classified	O67
Pregnancy or Puerperium			Labour and delivery complicated by fetal stress [distress]	O68
Pregnancy or Puerperium			Labour and delivery complicated by umbilical cord complications	O69
Pregnancy or Puerperium			Perineal laceration during delivery	O70
Pregnancy or Puerperium			Other obstetric trauma	O71
Pregnancy or Puerperium			Postpartum haemorrhage	O72
Pregnancy or Puerperium			Retained placenta and membranes, without haemorrhage	O73
Pregnancy or Puerperium			Complications of anaesthesia during labour and delivery	O74
Pregnancy or Puerperium			Other complications of labour and delivery, not elsewhere classified	O75
Pregnancy or Puerperium			Single spontaneous delivery	O80
Pregnancy or Puerperium			Single delivery by forceps and vacuum extractor	O81
Pregnancy or Puerperium			Single delivery by caesarean section	O82
Pregnancy or Puerperium			Other assisted single delivery	O83
Pregnancy or Puerperium			Multiple delivery	O84
Pregnancy or Puerperium			Puerperal sepsis	O85
Pregnancy or Puerperium			Other puerperal infections	O86

Status	ICD-9 disease classification	ICD-9 Code	ICD-10 disease classification	ICD-10 Code
Pregnancy or Puerperium			Venous complications in the puerperium	O87
Pregnancy or Puerperium			Obstetric embolism	O88
Pregnancy or Puerperium			Complications of anaesthesia during the puerperium	O89
Pregnancy or Puerperium			Complications of the puerperium, not elsewhere classified	O90
Pregnancy or Puerperium			Infections of breast associated with childbirth	O91
Pregnancy or Puerperium			Other disorders of breast and lactation associated with childbirth	O92
Pregnancy or Puerperium			Sequelae of complication of pregnancy, childbirth and the puerperium	O94
Pregnancy or Puerperium			Obstetric death of unspecified cause	O95
Pregnancy or Puerperium			Death from any obstetric cause occurring more than 42 days but less than one year after delivery	O96
Pregnancy or Puerperium			Death from sequelae of obstetric causes	O97
Pregnancy or Puerperium			Maternal infectious and parasitic diseases classifiable elsewhere but complicating pregnancy, childbirth and the puerperium	O98
Pregnancy or Puerperium			Other maternal diseases classifiable elsewhere but complicating pregnancy, childbirth and the puerperium	O99
Pregnancy or Puerperium			Pregnant state, incidental	Z33
Pregnancy or Puerperium			Supervision of normal pregnancy	Z34
Pregnancy or Puerperium			Supervision of high-risk pregnancy	Z35
Pregnancy or Puerperium			Antenatal screening	Z36
Pregnancy or Puerperium			Outcome of delivery	Z37
Pregnancy or Puerperium			Liveborn infants according to place of birth	Z38

Supplementary Table 2. Missing Data Situations and Filling Strategies

Variable	Missing number	Missing percentage (%)	Filling Strategy
Age in years at admission	35	< .01	28
Race	3844387	9.34	White
Control/ownership of hospital	222805	0.54	Private not for profit
Bed size of hospital	222805	0.54	Large
Teaching status of hospital	222805	0.54	Teaching
Location status of hospital	222805	0.54	Urban
Region of hospital	0	0	-
Primary expected payer	63421.25	0.15	Private insurance
Median household income percentile	660445.4	1.60	<25
In-hospital death	14017.09	0.03	Survival
Length of stay days	132.2377	< .01	2
Patient disposition	13741.88	0.03	Routine

Supplementary Table 3. Temporal Trends of the Incidence of Acute High-risk Chest Pain Diseases per 100,000 Patients During Pregnancy and Puerperium

Variable	2008	2009	2010	2011	2012	2013	2014	2015	2016	2017	Z Value	P Value
Acute high-risk chest pain diseases	79.92	95.95	95.96	91.97	98.52	89.49	100.17	105.49	108.7	114.79	-16.76	<0.0001
Pregnancy	67.03	94.18	97.57	82.32	101.41	118.76	135.22	83.76	23.59	25.48	23.01	<0.0001
Puerperium	81.05	96.11	95.81	92.78	98.29	87.33	97.71	108.64	147.13	155.59	-31.49	<0.0001
Pulmonary embolism	72.05	84.77	85.50	79.80	84.48	77.46	88.44	90.58	93.16	96.52	-12.45	<0.0001
Pregnancy	27.19	32.13	45.49	19.87	30.42	47.51	67.61	33.11	0.40	0.81	20.56	<0.0001
Puerperium	75.97	89.43	89.13	84.80	88.77	79.66	89.91	98.90	135.03	140.25	-28.40	<0.0001
Acute myocardial infarction	5.87	9.24	8.61	10.36	10.56	9.27	7.78	10.97	12.06	14.84	-11.66	<0.0001
Pregnancy	30.18	48.85	41.97	53.63	67.61	54.81	52.59	41.88	15.19	19.82	13.34	<0.0001
Puerperium	3.75	5.74	5.58	6.74	6.03	5.92	4.63	6.49	10.65	12.57	-12.71	<0.0001
Aortic dissection	2.21	2.61	2.48	2.43	3.48	3.26	4.32	4.93	3.98	4.31	-8.93	<0.0001
Pregnancy	9.66	13.20	10.11	8.83	3.38	16.44	15.02	9.74	8.00	4.85	4.13	<0.0001
Puerperium	1.56	1.68	1.79	1.90	3.49	2.29	3.57	4.23	2.17	4.07	-8.78	<0.0001

Supplementary Table 4. Temporal Trends of the Incidence of Subgroups Which Categorized by Age, Status, Race, Household income, Insurance type, and Region, per 100,000 Patients

Subgroups	Type	2008	2009	2010	2011	2012	2013	2014	2015	2016	2017	Z Value	P Value
Elderly Parturient	Non-elderly parturient	72.53	86	87.7	81.47	88.09	78.14	91.35	96.99	100.93	105.86	-16.17	<0.0001
Elderly Parturient	Elderly parturient	122.01	152.6	144.39	151.41	157.17	150.79	146.88	148.85	146.17	155.98	-3.13	0.0017
Status	Pregnancy	67.03	94.18	97.57	82.32	101.41	118.76	135.22	83.76	23.59	25.48	23.01	<0.0001
Status	Puerperium	81.05	96.11	95.81	92.78	98.29	87.33	97.71	108.64	147.13	155.59	-31.49	<0.0001
Race	White	78.97	99.3	93.11	91.62	88.93	88.49	100.43	101.77	104.16	110.04	-10.64	<0.0001
Race	Black	173.81	172.11	186.75	168.15	182.71	149.29	194.63	198.23	206.98	195.88	-5.30	<0.0001
Race	Hispanic	45.01	56.93	52.67	51.49	71.65	57.67	50.3	72.14	74.45	79.94	-10.16	<0.0001
Race	Asian or Pacific Islander	28.84	36.95	52.17	41.67	61.43	44.61	43.86	41.39	55.63	55.35	-3.55	0.0004
Race	Native American	18.12	50.18	42.79	151.5	146.05	125.16	73.92	141.82	51.44	180.6	-5.33	<0.0001
Race	Other	74.6	71.6	77.43	93.11	99.8	95.25	85.69	62.37	69.31	118.45	-2.30	0.0213
Household income	$<P_{25}$	79.88	107.7	112.96	101.66	100.57	101.58	124.73	129.77	139.77	140.47	-16.48	<0.0001
Household income	P_{25}~P_{49}	79.41	102.2	92.43	101.93	112.82	95.63	98.57	109	114.99	128.08	-10.33	<0.0001
Household income	P_{50}~P_{74}	84.67	93.34	90.92	87.26	98.54	80.78	83.02	97.03	92.63	82.78	0.32	0.7509
Household income	$\geq P_{75}$	75.66	77.02	82.51	74.27	79.9	75.91	87.34	77.2	77.27	100.08	-4.15	<0.0001
Insurance types	Medicare	129.91	534.5	244.44	330.39	291.02	373.02	310.74	251.57	313.03	247.68	0.02	0.9828
Insurance types	Medicaid	84.71	100.19	105.25	96.45	111.1	95.74	106.31	124.61	124.92	132.24	-15.77	<0.0001
Insurance types	Private insurance	72.95	83.37	80.95	86.18	85.09	77.53	88.99	88.33	90.08	96.93	-8.35	<0.0001
Insurance types	Self-pay	103.69	103.65	113.1	73.68	97.07	99	142.37	96.32	130.56	114.52	-2.13	0.0333
Insurance types	No charge	57.74	122.11	145.66	81.03	293.26	338.6	147.06	339.56	373.83	0	-3.23	0.0012
Insurance types	Other	100.07	135.46	140.2	75.36	73.83	103.11	100.97	84.78	105.84	131.48	0.66	0.5076
Hospital region	Northeast	85.19	90.56	107.43	99.27	109.13	92.2	104.41	108.34	109.65	128.5	-7.46	<0.0001
Hospital region	Midwest	86.81	111.29	105.98	113.68	111.44	104.44	115.2	119.09	124.7	130.07	-8.66	<0.0001
Hospital region	South	81.23	95.23	93.93	84.56	100.2	90.85	102.37	103.33	116.37	116.51	-12.16	<0.0001
Hospital region	West	68.45	86.75	82.78	80.46	77.25	72.47	80.71	95.13	81.87	89.21	-4.20	<0.0001

Supplementary Table 5. Temporal Trends of the In-Hospital Mortality of Acute High-risk Chest Pain Diseases During Pregnancy and Puerperium

Variable	2008	2009	2010	2011	2012	2013	2014	2015	2016	2017	Z Value	P Value
Acute high-risk chest pain	2.24	2.54	1.73	2.86	1.77	2.1	2.1	1.99	1.37	2.21	2.26	0.0240
Pulmonary embolism	2.19	1.96	1.39	2.85	1.32	2.27	1.96	1.63	0.93	2.37	1.40	0.1620
Acute myocardial infarction	5.51	8.42	5.46	3.53	4.71	1.35	3.17	6.74	2.06	2.56	3.90	<0.0001
Aortic dissection	0	0	5.27	4.77	3.57	0	2.86	0	9.38	0	-0.97	0.3318

Supplementary Table 6. Association of Hospitalization Cost and LOS with Acute High-risk Chest Pain Diseases During Pregnancy and Puerperium

Items	Estimate	95% Lower CL	95% Upper CL	P Value
Association of high-risk chest pain compared with no high-risk chest pain with each outcome among all participants				
Cost, dollars	35,789.000	35,402.000	36,177.000	<0.0001
LOS, days	2.488	2.432	2.543	<0.0001
Association of each outcome with unit increase in year with all patients				
Cost, dollars	536.643	532.319	540.966	<0.0001
LOS, days	-0.025	-0.025	-0.024	<0.0001
Association of each outcome with unit increase in year when restricted to participants with comorbid high-risk chest pain				
Cost, dollars	-58.811	-904.297	786.676	0.8915
LOS, days	-0.207	-0.266	-0.148	<0.0001
Association of each outcome with unit increase in year when restricted to participants without comorbid high-risk chest pain				
Cost, dollars	537.273	533.042	541.506	<0.0001
LOS, days	-0.025	-0.025	-0.024	<0.0001

Obtained from multivariate linear models with either cost or LOS as the dependent variable and acute high-risk chest pain as the predictor variable, adjusted for the same covariate assessment of model 1.

Supplementary Table 7. Association of In-hospital Death with Acute High-risk Chest Pain Diseases and Clinical Factors

Variables	Unadjusted Model		Multivariate Model 1		Multivariate Model 2		Multivariate Model 3	
	OR (95% CI)	P value	OR (95% CI)	P value	OR (95% CI)	P value	OR (95% CI)	P value
Acute high-risk chest pain	235.84(218.67-254.35)	<0.0001	56.10(51.26-61.39)	<0.0001	45.60(39.59-52.51)	<0.0001	57.42(52.43-62.87)	<0.0001
Age group								
18~24-25~29	1.09(1.00-1.18)	0.0447	1.10(1.01-1.20)	0.0289	1.21(1.10-1.33)	<0.0001	1.09(1.00-1.19)	0.0479
18~24-30~34	1.25(1.15-1.36)	<0.0001	1.32(1.21-1.45)	<0.0001	1.35(1.23-1.49)	<0.0001	1.31(1.20-1.43)	<0.0001
18~24-35~39	1.61(1.47-1.77)	<0.0001	1.51(1.36-1.67)	<0.0001	1.65(1.48-1.84)	<0.0001	1.50(1.35-1.66)	<0.0001
18~24-40~44	3.00(2.64-3.41)	<0.0001	2.30(2.00-2.64)	<0.0001	2.71(2.35-3.13)	<0.0001	2.26(1.97-2.60)	<0.0001
18~24-≥45	14.79(12.15-18.00)	<0.0001	7.76(6.24-9.66)	<0.0001	8.32(6.58-10.51)	<0.0001	7.43(5.94-9.29)	<0.0001
Race								
White-Black	2.87(2.68-3.07)	<0.0001	1.82(1.68-1.96)	<0.0001	2.10(1.94-2.29)	<0.0001	1.80(1.67-1.94)	<0.0001
White-Hispanic	0.99(0.91-1.08)	0.7862	0.90(0.82-0.99)	0.0291	0.97(0.88-1.08)	0.5693	0.85(0.77-0.94)	0.0016
White- Asian or Pacific Islander	1.36(1.19-1.56)	<0.0001	1.61(1.40-1.85)	<0.0001	1.75(1.51-2.03)	<0.0001	1.65(1.43-1.89)	<0.0001
White-Native America	2.56(2.00-3.27)	<0.0001	2.62(2.04-3.37)	<0.0001	2.64(2.00-3.47)	<0.0001	2.60(2.02-3.35)	<0.0001
White-Other	1.29(1.12-1.50)	0.0006	1.12(0.96-1.30)	0.1543	1.31(1.12-1.53)	0.0009	1.07(0.92-1.25)	0.3862
Primary expected pay								
Medicare-Medicaid	0.21(0.18-0.24)	<0.0001	0.63(0.54-0.74)	<0.0001	0.61(0.51-0.72)	<0.0001	0.62(0.53-0.73)	<0.0001
Medicare-Private ins	0.11(0.10-0.13)	<0.0001	0.39(0.33-0.46)	<0.0001	0.37(0.31-0.43)	<0.0001	0.38(0.32-0.45)	<0.0001
Medicare-Self-pay	0.32(0.27-0.39)	<0.0001	1.04(0.85-1.27)	0.7299	0.99(0.80-1.23)	0.9503	1.04(0.85-1.28)	0.6976
Medicare- No charge	0.59(0.38-0.92)	0.0186	1.53(0.97-2.42)	0.0675	1.07(0.62-1.82)	0.8171	1.54(0.97-2.43)	0.0654
Medicare- Other	0.17(0.14-0.21)	<0.0001	0.57(0.45-0.72)	<0.0001	0.53(0.41-0.68)	<0.0001	0.56(0.44-0.71)	<0.0001
Median household income								

Variables	Unadjusted Model		Multivariate Model 1		Multivariate Model 2		Multivariate Model 3	
	OR (95% CI)	P value	OR (95% CI)	P value	OR (95% CI)	P value	OR (95% CI)	P value
<25-25~49	0.84(0.78-0.90)	<0.0001	1.10(1.02-1.18)	0.0190	1.15(1.06-1.25)	0.0007	1.07(0.99-1.15)	0.1077
<25-50~74	0.62(0.57-0.67)	<0.0001	0.84(0.77-0.92)	0.0001	0.88(0.81-0.97)	0.0092	0.82(0.75-0.90)	<0.0001
<25-≥75	0.47(0.43-0.52)	<0.0001	0.68(0.62-0.76)	<0.0001	0.76(0.68-0.85)	<0.0001	0.67(0.60-0.74)	<0.0001
Control/ownership of hospital								
Government nonfederal- Private not for profit	0.77(0.71-0.84)	<0.0001	0.98(0.90-1.07)	0.7061	0.94(0.86-1.03)	0.2076	1.01(0.92-1.10)	0.8874
Government nonfederal- Private investor owned	0.71(0.64-0.79)	<0.0001	1.14(1.01-1.28)	0.0318	1.15(1.02-1.30)	0.0279	1.15(1.02-1.30)	0.0195
Bed size of hospital								
Small-Medium	2.07(1.81-2.37)	<0.0001	1.76(1.54-2.02)	<0.0001	1.67(1.45-1.94)	<0.0001	1.78(1.55-2.05)	<0.0001
Small-Large	2.85(2.51-3.24)	<0.0001	2.20(1.94-2.51)	<0.0001	2.10(1.83-2.41)	<0.0001	2.22(1.95-2.53)	<0.0001
Teaching hospital	2.23(2.08-2.38)	<0.0001	1.50(1.39-1.62)	<0.0001	1.32(1.22-1.44)	<0.0001	1.50(1.39-1.62)	<0.0001
Urban hospital	2.46(2.14-2.83)	<0.0001	1.51(1.29-1.76)	<0.0001	1.55(1.31-1.84)	<0.0001	1.51(1.29-1.77)	<0.0001
Region of hospital								
Northeast-Midwest	0.89(0.81-0.99)	0.0302	0.96(0.86-1.06)	0.4093	0.94(0.84-1.06)	0.3247	0.98(0.88-1.09)	0.6890
Northeast-South	1.29(1.18-1.40)	<0.0001	1.27(1.16-1.39)	<0.0001	1.29(1.17-1.43)	<0.0001	1.29(1.18-1.42)	<0.0001
Northeast-West	0.82(0.74-0.91)	0.0001	1.00(0.90-1.12)	0.9643	0.98(0.88-1.10)	0.7503	1.02(0.92-1.14)	0.7107
Gestational hypertension	2.83(2.65-3.03)	<0.0001	1.23(1.10-1.37)	0.0002	1.32(1.18-1.48)	<0.0001	1.27(1.14-1.41)	<0.0001
Pre-eclampsia/eclampsia	3.90(3.60-4.22)	<0.0001	1.78(1.57-2.03)	<0.0001	1.63(1.42-1.86)	<0.0001	1.71(1.50-1.94)	<0.0001
Gestational diabetes	1.84(1.60-2.11)	<0.0001	1.17(1.01-1.35)	0.0373	1.18(1.02-1.37)	0.0261	1.19(1.03-1.38)	0.0165
Multiple pregnancy	1.08(0.83-1.40)	0.5818	0.61(0.46-0.81)	0.0007	0.73(0.55-0.97)	0.0325	0.65(0.49-0.86)	0.0023

Variables	Unadjusted Model		Multivariate Model 1		Multivariate Model 2		Multivariate Model 3	
	OR (95% CI)	P value	OR (95% CI)	P value	OR (95% CI)	P value	OR (95% CI)	P value
Acquired immune deficiency syndrome	37.90(28.26-50.83)	<0.0001	9.37(6.80-12.92)	<0.0001	7.83(5.39-11.39)	<0.0001	10.29(7.54-14.05)	<0.0001
Alcohol abuse	4.90(3.52-6.83)	<0.0001						
Deficiency anemias	1.94(1.79-2.11)	<0.0001	1.43(1.28-1.60)	<0.0001	1.19(1.05-1.34)	0.0063	1.38(1.23-1.55)	<0.0001
Rheumatoid arthritis/collagen vascular diseases	6.43(5.21-7.94)	<0.0001	1.44(1.14-1.81)	0.0019	1.60(1.26-2.02)	<0.0001	1.43(1.14-1.80)	0.0023
Chronic blood loss anemia	1.90(1.76-2.04)	<0.0001	0.64(0.57-0.71)	<0.0001	0.81(0.73-0.91)	0.0002	0.67(0.60-0.74)	<0.0001
Chronic pulmonary disease	2.33(2.11-2.57)	<0.0001	1.21(1.09-1.35)	0.0005	1.28(1.14-1.44)	<0.0001	1.20(1.08-1.34)	0.0009
Coagulopathy	29.72(27.96-31.58)	<0.0001	15.84(14.79-16.95)	<0.0001	16.76(15.58-18.03)	<0.0001	16.03(14.97-17.17)	<0.0001
Depression	1.76(1.53-2.03)	<0.0001	0.76(0.65-0.88)	0.0004	0.84(0.71-0.98)	0.0294	0.79(0.68-0.92)	0.0030
Drug abuse	3.78(3.38-4.22)	<0.0001	1.69(1.49-1.92)	<0.0001	1.75(1.53-2.00)	<0.0001	1.70(1.50-1.93)	<0.0001
Hypothyroidism	1.37(1.17-1.60)	<0.0001						
Liver disease	13.26(11.13-15.79)	<0.0001	3.03(2.48-3.70)	<0.0001	3.50(2.86-4.29)	<0.0001	2.71(2.20-3.33)	<0.0001
Lymphoma	12.62(6.69-23.78)	<0.0001	4.01(2.08-7.74)	<0.0001	4.39(2.25-8.58)	<0.0001	3.96(2.04-7.67)	<0.0001
Metastatic cancer	249.51(200.83-310.00)	<0.0001	51.05(39.15-66.58)	<0.0001	56.61(42.66-75.11)	<0.0001	-	-
Other neurological disorders	22.04(20.37-23.84)	<0.0001	8.93(8.14-9.79)	<0.0001	8.71(7.89-9.62)	<0.0001	9.01(8.21-9.88)	<0.0001
Obesity	2.35(2.16-2.55)	<0.0001	1.25(1.14-1.37)	<0.0001	1.39(1.26-1.53)	<0.0001	1.24(1.13-1.36)	<0.0001
Paralysis	56.11(47.24-66.64)	<0.0001	4.45(3.60-5.49)	<0.0001	5.87(4.75-7.26)	<0.0001	4.71(3.81-5.83)	<0.0001
Psychoses	2.04(1.67-2.49)	<0.0001	0.63(0.51-0.78)	<0.0001	0.69(0.55-0.87)	0.0015	0.64(0.51-0.79)	<0.0001
Solid tumor without metastasis	31.32(22.83-42.97)	<0.0001	12.97(9.23-18.22)	<0.0001	11.50(7.96-16.60)	<0.0001	12.95(9.22-18.19)	<0.0001
Peptic ulcer disease excluding bleeding	44.02(23.65-81.95)	<0.0001	7.88(3.68-16.86)	<0.0001	10.45(5.36-20.39)	<0.0001	8.06(3.74-17.33)	<0.0001

Variables	Unadjusted Model		Multivariate Model 1		Multivariate Model 2		Multivariate Model 3	
	OR (95% CI)	P value	OR (95% CI)	P value	OR (95% CI)	P value	OR (95% CI)	P value
Valvular disease	15.52(13.52-17.82)	<0.0001	3.87(3.28-4.55)	<0.0001	4.88(4.12-5.78)	<0.0001	3.83(3.25-4.50)	<0.0001
Weight loss	67.83(59.90-76.81)	<0.0001	8.77(7.51-10.25)	<0.0001	11.70(9.98-13.71)	<0.0001	8.59(7.31-10.10)	<0.0001

Supplementary Table 8. Association of Pulmonary Circulation Disorders with Acute High-risk Chest Pain Diseases and Clinical Factors

Variables	Unadjusted Model		Multivariate Model 1		Multivariate Model 2	
	OR (95% CI)	P value	OR (95% CI)	P value	OR (95% CI)	P value
Acute high-risk chest pain	487.29(472.12-502.95)	<0.0001	255.96(246.32-265.97)	<0.0001	2066.12(1964.95-2172)	<0.0001
Age group						
18~24-25~29	1.24(1.19-1.28)	<0.0001	1.24(1.19-1.29)	<0.0001	1.27(1.22-1.33)	<0.0001
18~24-30~34	1.36(1.30-1.41)	<0.0001	1.31(1.25-1.37)	<0.0001	1.27(1.21-1.33)	<0.0001
18~24-35~39	1.85(1.77-1.93)	<0.0001	1.48(1.41-1.55)	<0.0001	1.42(1.34-1.50)	<0.0001
18~24-40~44	2.73(2.56-2.91)	<0.0001	1.62(1.51-1.74)	<0.0001	1.45(1.34-1.58)	<0.0001
18~24-≥45	6.21(5.39-7.15)	<0.0001	2.40(2.03-2.84)	<0.0001	1.34(1.09-1.65)	0.0048
Race						
White-Black	2.87(2.78-2.96)	<0.0001	1.76(1.70-1.83)	<0.0001	2.09(2.01-2.18)	<0.0001
White-Hispanic	0.89(0.85-0.93)	<0.0001	0.97(0.93-1.02)	0.2431	1.11(1.06-1.17)	<0.0001
White- Asian or Pacific Islander	0.84(0.78-0.91)	<0.0001	1.18(1.09-1.28)	<0.0001	1.31(1.20-1.43)	<0.0001
White-Native America	1.22(1.04-1.43)	0.0159	1.13(0.96-1.34)	0.1488	1.01(0.83-1.24)	0.9005
White-Other	1.18(1.10-1.26)	<0.0001	1.25(1.16-1.35)	<0.0001	1.49(1.37-1.61)	<0.0001
Primary expected pay						
Medicare-Medicaid	0.21(0.19-0.22)	<0.0001	0.50(0.46-0.53)	<0.0001	0.54(0.50-0.60)	<0.0001
Medicare-Private ins	0.12(0.12-0.13)	<0.0001	0.32(0.30-0.35)	<0.0001	0.37(0.33-0.40)	<0.0001
Medicare-Self-pay	0.17(0.15-0.19)	<0.0001	0.45(0.40-0.50)	<0.0001	0.48(0.42-0.55)	<0.0001
Medicare- No charge	0.46(0.37-0.58)	<0.0001	1.04(0.80-1.34)	0.7767	1.11(0.84-1.48)	0.4557
Medicare- Other	0.18(0.16-0.19)	<0.0001	0.46(0.41-0.52)	<0.0001	0.49(0.43-0.56)	<0.0001
Median household income						
<25-25~49	0.75(0.73-0.78)	<0.0001	0.93(0.89-0.97)	0.0002	0.96(0.92-1.00)	0.0766
<25-50~74	0.66(0.64-0.68)	<0.0001	0.86(0.82-0.89)	<0.0001	0.91(0.86-0.95)	<0.0001
<25-≥75	0.60(0.57-0.62)	<0.0001	0.86(0.82-0.90)	<0.0001	0.93(0.88-0.98)	0.0042
Control/ownership of hospital						
Government nonfederal- Private not for profit	0.73(0.71-0.76)	<0.0001	0.82(0.78-0.85)	<0.0001	0.80(0.77-0.84)	<0.0001
Government nonfederal- Private investor owned	0.52(0.50-0.55)	<0.0001	0.75(0.71-0.80)	<0.0001	0.74(0.70-0.79)	<0.0001
Bed size of hospital						
Small-Medium	1.39(1.32-1.47)	<0.0001	1.24(1.16-1.31)	<0.0001	1.24(1.16-1.32)	<0.0001
Small-Large	1.94(1.84-2.04)	<0.0001	1.50(1.42-1.58)	<0.0001	1.41(1.33-1.49)	<0.0001
Teaching hospital	1.86(1.81-1.92)	<0.0001	1.18(1.14-1.22)	<0.0001	1.07(1.03-1.11)	0.0012
Urban hospital	2.53(2.37-2.70)	<0.0001	1.77(1.64-1.91)	<0.0001	1.75(1.61-1.91)	<0.0001
Region of hospital						
Northeast-Midwest	0.99(0.94-1.03)	0.5372				

Variables	Unadjusted Model		Multivariate Model 1		Multivariate Model 2	
	OR (95% CI)	P value	OR (95% CI)	P value	OR (95% CI)	P value
Northeast-South	1.06(1.02-1.10)	0.0021				
Northeast-West	0.75(0.72-0.79)	<0.0001				
Gestational hypertension	4.79(4.66-4.93)	<0.0001	3.15(3.06-3.26)	<0.0001	3.59(3.44-3.75)	<0.0001
Pre-eclampsia/eclampsia	3.77(3.63-3.92)	<0.0001			0.91(0.86-0.97)	0.0015
Gestational diabetes	2.09(1.97-2.22)	<0.0001	1.20(1.12-1.28)	<0.0001	1.22(1.14-1.32)	<0.0001
Multiple pregnancy	0.87(0.76-1.00)	0.0508	0.58(0.50-0.67)	<0.0001	0.58(0.49-0.68)	<0.0001
Acquired immune deficiency syndrome	11.75(9.21-14.99)	<0.0001	3.07(2.32-4.05)	<0.0001	3.64(2.65-5.00)	<0.0001
Alcohol abuse	2.95(2.42-3.59)	<0.0001				
Deficiency anemias	3.80(3.69-3.92)	<0.0001	1.91(1.81-2.01)	<0.0001	1.73(1.64-1.83)	<0.0001
Rheumatoid arthritis/collagen vascular diseases	11.56(10.73-12.46)	<0.0001	3.49(3.20-3.82)	<0.0001	3.53(3.18-3.91)	<0.0001
Chronic blood loss anemia	3.61(3.51-3.72)	<0.0001	1.21(1.16-1.27)	<0.0001	1.47(1.39-1.55)	<0.0001
Chronic pulmonary disease	4.45(4.29-4.62)	<0.0001	2.08(1.99-2.17)	<0.0001	2.27(2.17-2.38)	<0.0001
Coagulopathy	5.26(5.02-5.52)	<0.0001	1.76(1.67-1.87)	<0.0001	1.88(1.76-2.01)	<0.0001
Depression	2.81(2.66-2.97)	<0.0001	1.25(1.18-1.33)	<0.0001	1.28(1.20-1.38)	<0.0001
Drug abuse	3.10(2.93-3.28)	<0.0001	1.34(1.25-1.43)	<0.0001	1.25(1.16-1.35)	<0.0001
Hypothyroidism	1.70(1.59-1.81)	<0.0001	1.15(1.07-1.24)	0.0001	1.16(1.07-1.26)	0.0003
Liver disease	7.25(6.50-8.08)	<0.0001	2.10(1.84-2.40)	<0.0001	2.38(2.05-2.76)	<0.0001
Lymphoma	7.24(4.90-10.68)	<0.0001	1.77(1.15-2.74)	0.0101		
Metastatic cancer	27.66(20.62-37.12)	<0.0001	4.20(2.85-6.19)	<0.0001	2.11(1.25-3.55)	0.0050
Other neurological disorders	4.20(3.91-4.52)	<0.0001	1.27(1.17-1.39)	<0.0001		
Obesity	3.96(3.83-4.10)	<0.0001	1.84(1.77-1.91)	<0.0001	1.79(1.71-1.87)	<0.0001
Paralysis	11.72(9.89-13.91)	<0.0001	1.80(1.47-2.21)	<0.0001	1.73(1.36-2.21)	<0.0001
Psychoses	2.79(2.57-3.02)	<0.0001				
Solid tumor without metastasis	7.70(5.74-10.34)	<0.0001	4.15(2.90-5.92)	<0.0001	4.61(3.02-7.03)	<0.0001
Peptic ulcer disease excluding bleeding	-	0.8275				
Valvular disease	117.28(113.67-121.00	<0.0001	76.49(73.65-79.43)	<0.0001	92.33(88.65-96.16)	<0.0001
Weight loss	22.14(20.09-24.41)	<0.0001	3.43(3.03-3.90)	<0.0001	3.03(2.58-3.57)	<0.0001

Supplementary Table 9. Association of Congestive Heart Failure with Acute High-risk Chest Pain Diseases and Clinical Factors

Variables	Unadjusted Model		Multivariate Model 1		Multivariate Model 2	
	OR (95% CI)	P value	OR (95% CI)	P value	OR (95% CI)	P value
Acute high-risk chest pain	52.48(50.25-54.80)	<0.0001	13.80(13.09-14.56)	<0.0001	22.86(21.18-24.66)	<0.0001
Age group						
18~24-25~29	1.24(1.21-1.27)	<0.0001	1.36(1.32-1.40)	<0.0001	1.38(1.33-1.42)	<0.0001
18~24-30~34	1.65(1.61-1.70)	<0.0001	1.90(1.84-1.95)	<0.0001	1.90(1.85-1.96)	<0.0001
18~24-35~39	2.66(2.59-2.74)	<0.0001	2.76(2.68-2.85)	<0.0001	2.84(2.75-2.94)	<0.0001
18~24-40~44	4.44(4.27-4.61)	<0.0001	3.81(3.66-3.97)	<0.0001	3.92(3.76-4.10)	<0.0001
18~24-≥45	14.88(13.91-15.92)	<0.0001	11.02(10.22-11.89)	<0.0001	11.23(10.37-12.16)	<0.0001
Race						
White-Black	3.62(3.55-3.69)	<0.0001	2.25(2.20-2.30)	<0.0001	2.45(2.40-2.51)	<0.0001
White-Hispanic	0.67(0.65-0.69)	<0.0001	0.68(0.65-0.70)	<0.0001	0.71(0.69-0.74)	<0.0001
White- Asian or Pacific Islander	0.77(0.73-0.81)	<0.0001	1.01(0.96-1.07)	0.6903	1.05(0.99-1.11)	0.0777
White-Native America	1.72(1.57-1.88)	<0.0001	1.49(1.35-1.64)	<0.0001	1.63(1.48-1.80)	<0.0001
White-Other	1.08(1.03-1.13)	0.0018	1.12(1.07-1.18)	<0.0001	1.20(1.14-1.27)	<0.0001
Primary expected pay						
Medicare-Medicaid	0.13(0.13-0.14)	<0.0001	0.32(0.30-0.33)	<0.0001	0.33(0.31-0.34)	<0.0001
Medicare-Private ins	0.08(0.08-0.08)	<0.0001	0.21(0.20-0.21)	<0.0001	0.21(0.21-0.22)	<0.0001
Medicare-Self-pay	0.12(0.11-0.12)	<0.0001	0.34(0.32-0.36)	<0.0001	0.36(0.34-0.39)	<0.0001
Medicare- No charge	0.12(0.10-0.15)	<0.0001	0.27(0.21-0.35)	<0.0001	0.32(0.25-0.40)	<0.0001
Medicare- Other	0.10(0.09-0.10)	<0.0001	0.27(0.25-0.29)	<0.0001	0.28(0.26-0.30)	<0.0001
Median household income						
<25-25~49	0.74(0.72-0.75)	<0.0001	0.94(0.92-0.96)	<0.0001	0.94(0.92-0.97)	<0.0001
<25-50~74	0.60(0.58-0.61)	<0.0001	0.79(0.77-0.81)	<0.0001	0.80(0.78-0.82)	<0.0001
<25-≥75	0.46(0.44-0.47)	<0.0001	0.63(0.61-0.65)	<0.0001	0.65(0.62-0.67)	<0.0001
Control/ownership of hospital						
Government nonfederal- Private not for profit	0.85(0.83-0.88)	<0.0001	1.01(0.98-1.03)	0.6886	1.00(0.97-1.03)	0.8935
Government nonfederal- Private investor owned	0.74(0.72-0.77)	<0.0001	1.06(1.02-1.10)	0.0030	1.05(1.01-1.09)	0.0074
Bed size of hospital						
Small-Medium	1.31(1.26-1.35)	<0.0001	1.16(1.12-1.20)	<0.0001	1.16(1.12-1.21)	<0.0001
Small-Large	1.75(1.70-1.81)	<0.0001	1.41(1.37-1.46)	<0.0001	1.37(1.33-1.42)	<0.0001
Teaching hospital	1.65(1.62-1.69)	<0.0001	1.06(1.04-1.09)	<0.0001		
Urban hospital	1.87(1.80-1.94)	<0.0001	1.47(1.40-1.53)	<0.0001	1.43(1.37-1.49)	<0.0001
Region of hospital						
Northeast-Midwest	1.21(1.18-1.25)	<0.0001	1.30(1.26-1.35)	<0.0001	1.26(1.22-1.30)	<0.0001

Variables	Unadjusted Model		Multivariate Model 1		Multivariate Model 2	
	OR (95% CI)	P value	OR (95% CI)	P value	OR (95% CI)	P value
Northeast-South	1.48(1.44-1.52)	<0.0001	1.41(1.37-1.45)	<0.0001	1.36(1.32-1.40)	<0.0001
Northeast-West	0.80(0.77-0.83)	<0.0001	1.11(1.07-1.15)	<0.0001	1.10(1.06-1.14)	<0.0001
Gestational hypertension	6.10(5.99-6.21)	<0.0001	3.31(3.23-3.39)	<0.0001	3.23(3.15-3.31)	<0.0001
Pre-eclampsia/eclampsia	5.10(4.99-5.22)	<0.0001	1.25(1.21-1.28)	<0.0001	1.26(1.22-1.30)	<0.0001
Gestational diabetes	2.55(2.45-2.64)	<0.0001	1.15(1.10-1.19)	<0.0001	1.12(1.08-1.17)	<0.0001
Multiple pregnancy	0.72(0.65-0.80)	<0.0001	0.42(0.38-0.47)	<0.0001	0.41(0.37-0.45)	<0.0001
Acquired immune deficiency syndrome	4.58(3.54-5.91)	<0.0001				
Alcohol abuse	5.20(4.71-5.74)	<0.0001	1.71(1.54-1.91)	<0.0001	1.75(1.56-1.97)	<0.0001
Deficiency anemias	4.27(4.18-4.35)	<0.0001	2.42(2.34-2.50)	<0.0001	2.49(2.40-2.58)	<0.0001
Rheumatoid arthritis/collagen vascular diseases	7.53(7.09-8.00)	<0.0001	2.32(2.17-2.48)	<0.0001	2.27(2.11-2.44)	<0.0001
Chronic blood loss anemia	3.67(3.60-3.74)	<0.0001	1.20(1.16-1.24)	<0.0001	1.16(1.12-1.20)	<0.0001
Chronic pulmonary disease	3.90(3.81-4.00)	<0.0001	1.84(1.79-1.89)	<0.0001	1.88(1.83-1.94)	<0.0001
Coagulopathy	3.37(3.24-3.50)	<0.0001	1.64(1.57-1.71)	<0.0001	1.70(1.62-1.77)	<0.0001
Depression	3.08(2.97-3.18)	<0.0001	1.51(1.46-1.57)	<0.0001	1.55(1.49-1.61)	<0.0001
Drug abuse	3.19(3.07-3.31)	<0.0001	1.54(1.47-1.60)	<0.0001	1.57(1.50-1.64)	<0.0001
Hypothyroidism	1.96(1.88-2.04)	<0.0001	1.35(1.29-1.41)	<0.0001	1.37(1.31-1.43)	<0.0001
Liver disease	6.46(5.99-6.97)	<0.0001	2.37(2.17-2.58)	<0.0001	2.49(2.28-2.73)	<0.0001
Lymphoma	15.92(13.36-18.96)	<0.0001	6.60(5.39-8.08)	<0.0001	6.17(4.96-7.68)	<0.0001
Metastatic cancer	6.57(4.43-9.73)	<0.0001	1.61(1.06-2.44)	0.0258		
Other neurological disorders	4.58(4.38-4.80)	<0.0001	1.66(1.57-1.74)	<0.0001	1.72(1.63-1.82)	<0.0001
Obesity	4.68(4.58-4.78)	<0.0001	2.18(2.13-2.24)	<0.0001	2.22(2.16-2.27)	<0.0001
Paralysis	13.80(12.43-15.32)	<0.0001	2.95(2.62-3.32)	<0.0001	3.07(2.70-3.48)	<0.0001
Psychoses	3.13(2.97-3.29)	<0.0001				
Solid tumor without metastasis	2.55(1.82-3.56)	<0.0001				
Peptic ulcer disease excluding bleeding	12.36(8.62-17.72)	<0.0001	4.47(3.04-6.56)	<0.0001	4.71(3.20-6.94)	<0.0001
Valvular disease	88.48(86.49-90.51)	<0.0001	57.14(55.70-58.62)	<0.0001	57.06(55.52-58.64)	<0.0001
Weight loss	22.45(21.05-23.94)	<0.0001	6.66(6.16-7.19)	<0.0001	6.39(5.88-6.95)	<0.0001

Supplementary Table 10. Association of Renal Failure with Acute High-risk Chest Pain Diseases and Clinical Factors

Variables	Unadjusted Model		Multivariate Model 1		Multivariate Model 2	
	OR (95% CI)	P value	OR (95% CI)	P value	OR (95% CI)	P value
Acute high-risk chest pain	9.64(8.67-10.73)	<0.0001	2.80(2.50-3.14)	<0.0001	5.30(4.56-6.15)	<0.0001
Age group						
18~24-25~29	1.25(1.22-1.29)	<0.0001	1.27(1.23-1.31)	<0.0001	1.26(1.22-1.30)	<0.0001
18~24-30~34	1.48(1.44-1.53)	<0.0001	1.44(1.39-1.48)	<0.0001	1.40(1.36-1.45)	<0.0001
18~24-35~39	2.01(1.95-2.08)	<0.0001	1.65(1.59-1.70)	<0.0001	1.62(1.56-1.68)	<0.0001
18~24-40~44	2.77(2.64-2.91)	<0.0001	1.81(1.72-1.91)	<0.0001	1.81(1.72-1.91)	<0.0001
18~24-≥45	9.32(8.51-10.20)	<0.0001	5.03(4.57-5.53)	<0.0001	4.79(4.33-5.31)	<0.0001
Elderly parturient women	1.81(1.77-1.86)	<0.0001				
Race						
White-Black	2.90(2.83-2.97)	<0.0001	1.60(1.56-1.65)	<0.0001	1.61(1.56-1.65)	<0.0001
White-Hispanic	1.33(1.29-1.36)	<0.0001	1.23(1.19-1.27)	<0.0001	1.25(1.21-1.29)	<0.0001
White- Asian or Pacific Islander	0.99(0.94-1.05)	0.8238	1.04(0.98-1.10)	0.1936	1.06(1.00-1.12)	0.0487
White-Native America	2.41(2.20-2.64)	<0.0001	1.89(1.72-2.08)	<0.0001	1.93(1.75-2.13)	<0.0001
White-Other	1.01(0.95-1.07)	0.8255	0.97(0.92-1.03)	0.3521	0.98(0.92-1.04)	0.4740
Primary expected pay						
Medicare-Medicaid	0.06(0.06-0.06)	<0.0001	0.12(0.12-0.13)	<0.0001	0.12(0.11-0.12)	<0.0001
Medicare-Private ins	0.04(0.04-0.04)	<0.0001	0.09(0.09-0.10)	<0.0001	0.09(0.09-0.10)	<0.0001
Medicare-Self-pay	0.06(0.06-0.07)	<0.0001	0.15(0.14-0.16)	<0.0001	0.15(0.14-0.16)	<0.0001
Medicare- No charge	0.04(0.03-0.06)	<0.0001	0.07(0.05-0.10)	<0.0001	0.07(0.05-0.10)	<0.0001
Medicare- Other	0.05(0.04-0.05)	<0.0001	0.11(0.10-0.12)	<0.0001	0.11(0.10-0.12)	<0.0001
Median household income						
<25-25~49	0.78(0.75-0.80)	<0.0001	1.00(0.97-1.02)	0.8025	1.00(0.97-1.03)	0.9022
<25-50~74	0.69(0.67-0.71)	<0.0001	0.92(0.89-0.95)	<0.0001	0.92(0.89-0.95)	<0.0001
<25-≥75	0.58(0.56-0.60)	<0.0001	0.88(0.85-0.91)	<0.0001	0.89(0.86-0.92)	<0.0001
Control/ownership of hospital						
Government nonfederal- Private not for profit	0.76(0.74-0.79)	<0.0001	0.93(0.90-0.95)	<0.0001	0.93(0.91-0.96)	<0.0001
Government nonfederal- Private investor owned	0.42(0.40-0.44)	<0.0001	0.68(0.65-0.71)	<0.0001	0.68(0.65-0.71)	<0.0001
Bed size of hospital						
Small-Medium	1.17(1.12-1.22)	<0.0001	1.15(1.11-1.20)	<0.0001	1.17(1.12-1.22)	<0.0001
Small-Large	1.92(1.85-1.99)	<0.0001	1.69(1.62-1.75)	<0.0001	1.70(1.63-1.77)	<0.0001
Teaching hospital	2.67(2.61-2.74)	<0.0001	1.74(1.69-1.79)	<0.0001	1.75(1.70-1.80)	<0.0001
Urban hospital	2.87(2.72-3.02)	<0.0001	1.60(1.51-1.70)	<0.0001	1.58(1.49-1.68)	<0.0001
Region of hospital						
Northeast-Midwest	1.22(1.18-1.26)	<0.0001	1.32(1.27-1.37)	<0.0001	1.34(1.29-1.39)	<0.0001

Variables	Unadjusted Model		Multivariate Model 1		Multivariate Model 2	
	OR (95% CI)	P value	OR (95% CI)	P value	OR (95% CI)	P value
Northeast-South	1.22(1.18-1.26)	<0.0001	1.22(1.18-1.26)	<0.0001	1.22(1.17-1.26)	<0.0001
Northeast-West	1.05(1.01-1.08)	0.0083	1.28(1.23-1.33)	<0.0001	1.26(1.21-1.31)	<0.0001
Gestational hypertension	7.70(7.54-7.86)	<0.0001	5.24(5.11-5.37)	<0.0001	5.09(4.96-5.23)	<0.0001
Pre-eclampsia/eclampsia	5.19(5.06-5.32)	<0.0001	0.89(0.86-0.92)	<0.0001	0.89(0.86-0.92)	<0.0001
Gestational diabetes	6.55(6.36-6.74)	<0.0001	3.06(2.97-3.16)	<0.0001	2.93(2.83-3.02)	<0.0001
Multiple pregnancy	1.80(1.67-1.93)	<0.0001				
Acquired immune deficiency syndrome	12.46(10.42-14.91)	<0.0001	2.88(2.38-3.48)	<0.0001	2.87(2.34-3.52)	<0.0001
Alcohol abuse	3.32(2.88-3.82)	<0.0001	1.21(1.04-1.40)	0.0124		
Deficiency anemias	6.16(6.03-6.30)	<0.0001	4.50(4.40-4.60)	<0.0001	4.50(4.40-4.61)	<0.0001
Rheumatoid arthritis/collagen vascular diseases	24.41(23.44-25.42)	<0.0001	8.48(8.10-8.87)	<0.0001	8.38(7.98-8.79)	<0.0001
Chronic blood loss anemia	4.62(4.52-4.71)	<0.0001				
Chronic pulmonary disease	2.49(2.41-2.58)	<0.0001	1.18(1.14-1.22)	<0.0001	1.18(1.14-1.23)	<0.0001
Coagulopathy	4.59(4.42-4.77)	<0.0001	2.15(2.07-2.24)	<0.0001	2.19(2.09-2.28)	<0.0001
Depression	3.25(3.12-3.37)	<0.0001	1.54(1.48-1.60)	<0.0001	1.57(1.50-1.64)	<0.0001
Drug abuse	2.46(2.34-2.58)	<0.0001	1.28(1.21-1.34)	<0.0001	1.33(1.27-1.41)	<0.0001
Hypothyroidism	2.60(2.49-2.70)	<0.0001	1.67(1.60-1.75)	<0.0001	1.74(1.66-1.82)	<0.0001
Liver disease	7.76(7.17-8.40)	<0.0001	2.81(2.58-3.07)	<0.0001	2.64(2.41-2.90)	<0.0001
Lymphoma	2.31(1.38-3.88)	0.0015				
Metastatic cancer	8.64(5.83-12.81)	<0.0001	2.12(1.39-3.23)	0.0005	2.29(1.50-3.49)	0.0001
Other neurological disorders	5.03(4.78-5.29)	<0.0001	1.76(1.67-1.86)	<0.0001	1.73(1.63-1.83)	<0.0001
Obesity	3.14(3.06-3.22)	<0.0001	1.19(1.16-1.22)	<0.0001	1.19(1.16-1.23)	<0.0001
Paralysis	7.73(6.60-9.05)	<0.0001	1.35(1.14-1.60)	0.0005	1.38(1.15-1.65)	0.0005
Psychoses	2.70(2.54-2.87)	<0.0001	0.77(0.72-0.82)	<0.0001	0.79(0.74-0.85)	<0.0001
Solid tumor without metastasis	1.89(1.21-2.95)	0.0052				
Peptic ulcer disease excluding bleeding	5.38(2.89-10.02)	<0.0001				
Valvular disease	6.88(6.41-7.39)	<0.0001	2.95(2.73-3.19)	<0.0001	2.79(2.57-3.03)	<0.0001
Weight loss	15.69(14.38-17.12)	<0.0001	4.82(4.38-5.30)	<0.0001	4.52(4.08-5.01)	<0.0001

Supplementary Table 11. Association of Fluid and Electrolyte Disorders with Acute High-risk Chest Pain Diseases and Clinical Factors

Variables	Unadjusted Model		Multivariate Model 1		Multivariate Model 2	
	OR (95% CI)	P value	OR (95% CI)	P value	OR (95% CI)	P value
Acute high-risk chest pain	16.00(15.58-16.43)	<0.0001	8.06(7.82-8.30)	<0.0001	10.32(9.80-10.87)	<0.0001
Age group						
18~24-25~29	0.77(0.77-0.78)	<0.0001	0.86(0.86-0.87)	<0.0001	0.86(0.86-0.87)	<0.0001
18~24-30~34	0.65(0.65-0.66)	<0.0001	0.78(0.77-0.78)	<0.0001	0.77(0.77-0.78)	<0.0001
18~24-35~39	0.69(0.68-0.69)	<0.0001	0.78(0.78-0.79)	<0.0001	0.79(0.78-0.79)	<0.0001
18~24-40~44	0.85(0.83-0.86)	<0.0001	0.87(0.86-0.89)	<0.0001	0.87(0.86-0.89)	<0.0001
18~24-≥45	1.66(1.59-1.73)	<0.0001	1.48(1.41-1.54)	<0.0001	1.50(1.43-1.58)	<0.0001
Race						
White-Black	2.22(2.21-2.24)	<0.0001	1.61(1.60-1.62)	<0.0001	1.63(1.62-1.64)	<0.0001
White-Hispanic	0.97(0.96-0.98)	<0.0001	0.89(0.89-0.90)	<0.0001	0.90(0.89-0.91)	<0.0001
White- Asian or Pacific Islander	0.71(0.70-0.72)	<0.0001	0.84(0.83-0.86)	<0.0001	0.84(0.83-0.86)	<0.0001
White-Native America	1.58(1.53-1.62)	<0.0001	1.23(1.19-1.26)	<0.0001	1.23(1.20-1.27)	<0.0001
White-Other	1.03(1.01-1.04)	0.0003	1.00(0.99-1.02)	0.7998	1.02(1.00-1.04)	0.0131
Primary expected pay						
Medicare-Medicaid	0.37(0.37-0.38)	<0.0001	0.57(0.56-0.59)	<0.0001	0.57(0.56-0.58)	<0.0001
Medicare-Private ins	0.22(0.22-0.23)	<0.0001	0.45(0.45-0.46)	<0.0001	0.46(0.45-0.47)	<0.0001
Medicare-Self-pay	0.47(0.46-0.48)	<0.0001	0.89(0.86-0.91)	<0.0001	0.93(0.91-0.96)	<0.0001
Medicare- No charge	0.59(0.56-0.62)	<0.0001	1.02(0.96-1.08)	0.5686	1.01(0.95-1.07)	0.7480
Medicare- Other	0.32(0.31-0.33)	<0.0001	0.57(0.56-0.59)	<0.0001	0.58(0.56-0.60)	<0.0001
Median household income						
<25-25~49	0.78(0.77-0.78)	<0.0001	0.93(0.92-0.93)	<0.0001	0.93(0.92-0.93)	<0.0001
<25-50~74	0.67(0.66-0.67)	<0.0001	0.88(0.87-0.88)	<0.0001	0.88(0.87-0.89)	<0.0001
<25-≥75	0.54(0.53-0.54)	<0.0001	0.81(0.80-0.82)	<0.0001	0.83(0.82-0.83)	<0.0001
Control/ownership of hospital						
Government nonfederal- Private not for profit	0.90(0.89-0.90)	<0.0001	1.07(1.06-1.08)	<0.0001	1.05(1.04-1.06)	<0.0001
Government nonfederal- Private investor owned	0.98(0.96-0.99)	<0.0001	1.17(1.16-1.18)	<0.0001	1.16(1.15-1.18)	<0.0001
Bed size of hospital						
Small-Medium	1.02(1.01-1.03)	<0.0001	0.99(0.98-1.00)	0.1782	0.99(0.98-1.00)	0.1154
Small-Large	1.15(1.14-1.16)	<0.0001	1.06(1.05-1.07)	<0.0001	1.05(1.04-1.06)	<0.0001
Teaching hospital	1.16(1.15-1.16)	<0.0001			0.98(0.98-0.99)	<0.0001
Urban hospital	0.98(0.98-0.99)	0.0009	0.98(0.97-0.99)	0.0004	0.98(0.96-0.99)	<0.0001
Region of hospital						
Northeast-Midwest	1.16(1.15-1.17)	<0.0001	1.12(1.11-1.14)	<0.0001	1.09(1.08-1.10)	<0.0001

Variables	Unadjusted Model		Multivariate Model 1		Multivariate Model 2	
	OR (95% CI)	P value	OR (95% CI)	P value	OR (95% CI)	P value
Northeast-South	1.34(1.33-1.35)	<0.0001	1.21(1.20-1.22)	<0.0001	1.18(1.17-1.19)	<0.0001
Northeast-West	0.99(0.99-1.00)	0.2739	1.11(1.10-1.12)	<0.0001	1.10(1.08-1.11)	<0.0001
Gestational hypertension	2.18(2.17-2.20)	<0.0001	1.40(1.39-1.42)	<0.0001	1.40(1.38-1.41)	<0.0001
Pre-eclampsia/eclampsia	2.71(2.68-2.73)	<0.0001	1.60(1.58-1.62)	<0.0001	1.60(1.58-1.62)	<0.0001
Gestational diabetes	1.93(1.91-1.96)	<0.0001	1.64(1.62-1.67)	<0.0001	1.62(1.60-1.64)	<0.0001
Multiple pregnancy	1.04(1.02-1.07)	0.0017	0.85(0.83-0.87)	<0.0001	0.86(0.84-0.89)	<0.0001
Acquired immune deficiency syndrome	4.93(4.55-5.34)	<0.0001	1.69(1.55-1.84)	<0.0001	1.63(1.48-1.80)	<0.0001
Alcohol abuse	6.07(5.89-6.26)	<0.0001	2.40(2.32-2.48)	<0.0001	2.37(2.29-2.45)	<0.0001
Deficiency anemias	3.02(3.00-3.04)	<0.0001	1.38(1.37-1.40)	<0.0001	1.39(1.37-1.40)	<0.0001
Rheumatoid arthritis/collagen vascular diseases	3.77(3.67-3.87)	<0.0001	2.21(2.15-2.28)	<0.0001	2.28(2.21-2.35)	<0.0001
Chronic blood loss anemia	3.07(3.06-3.09)	<0.0001	1.91(1.89-1.93)	<0.0001	1.90(1.88-1.92)	<0.0001
Chronic pulmonary disease	2.46(2.44-2.48)	<0.0001	1.68(1.66-1.70)	<0.0001	1.68(1.66-1.70)	<0.0001
Coagulopathy	3.92(3.87-3.96)	<0.0001	2.78(2.75-2.82)	<0.0001	2.82(2.79-2.86)	<0.0001
Depression	2.37(2.34-2.40)	<0.0001	1.61(1.59-1.63)	<0.0001	1.62(1.60-1.65)	<0.0001
Drug abuse	3.21(3.18-3.25)	<0.0001	1.80(1.77-1.82)	<0.0001	1.83(1.80-1.85)	<0.0001
Hypothyroidism	1.22(1.20-1.24)	<0.0001	1.24(1.22-1.26)	<0.0001	1.24(1.22-1.26)	<0.0001
Liver disease	4.55(4.42-4.69)	<0.0001	2.50(2.42-2.58)	<0.0001	2.51(2.43-2.60)	<0.0001
Lymphoma	4.81(4.34-5.33)	<0.0001	3.06(2.73-3.42)	<0.0001	3.22(2.86-3.63)	<0.0001
Metastatic cancer	13.89(12.61-15.31)	<0.0001	6.55(5.83-7.36)	<0.0001	7.15(6.32-8.08)	<0.0001
Other neurological disorders	4.34(4.27-4.41)	<0.0001	2.50(2.46-2.54)	<0.0001	2.47(2.43-2.52)	<0.0001
Obesity	1.47(1.46-1.48)	<0.0001	0.94(0.93-0.95)	<0.0001	0.95(0.94-0.96)	<0.0001
Paralysis	8.23(7.87-8.61)	<0.0001	3.05(2.90-3.21)	<0.0001	3.12(2.95-3.29)	<0.0001
Psychoses	3.39(3.34-3.45)	<0.0001	1.72(1.69-1.75)	<0.0001	1.71(1.68-1.74)	<0.0001
Solid tumor without metastasis	4.34(3.99-4.72)	<0.0001	3.30(3.02-3.61)	<0.0001	3.03(2.74-3.34)	<0.0001
Peptic ulcer disease excluding bleeding	12.01(10.57-13.65)	<0.0001	5.50(4.74-6.39)	<0.0001	5.01(4.27-5.87)	<0.0001
Valvular disease	5.41(5.29-5.54)	<0.0001	3.93(3.84-4.03)	<0.0001	3.97(3.87-4.08)	<0.0001
Weight loss	57.92(56.74-59.13)	<0.0001	37.22(36.39-38.08)	<0.0001	37.34(36.45-38.24)	<0.0001

Supplementary Table 12. Association of Clinical Factors with Aortic Dissection During Pregnancy and Puerperium

Variables	Unadjusted Model		Multivariate Model 1		Multivariate Model 2	
	OR (95% CI)	P value	OR (95% CI)	P value	OR (95% CI)	P value
Age group						
18~24-25~29	1.30(1.10-1.55)	0.0024	1.17(0.98-1.39)	0.0803	1.05(0.88-1.27)	0.5717
18~24-30~34	1.96(1.67-2.30)	<0.0001	1.63(1.38-1.93)	<0.0001	1.51(1.27-1.81)	<0.0001
18~24-35~39	3.53(2.99-4.17)	<0.0001	2.60(2.18-3.09)	<0.0001	2.29(1.90-2.75)	<0.0001
18~24-40~44	3.09(2.36-4.04)	<0.0001	1.92(1.46-2.54)	<0.0001	1.70(1.27-2.29)	0.0004
18~24-≥45	18.07(12.30-26.53)	<0.0001	7.55(5.06-11.26)	<0.0001	7.78(5.20-11.64)	<0.0001
Race						
White-Black	0.79(0.68-0.92)	0.0033	0.53(0.45-0.63)	<0.0001	0.52(0.44-0.62)	<0.0001
White-Hispanic	0.46(0.39-0.54)	<0.0001	0.49(0.41-0.59)	<0.0001	0.50(0.41-0.60)	<0.0001
White- Asian or Pacific Islander	0.24(0.15-0.37)	<0.0001	0.27(0.17-0.42)	<0.0001	0.27(0.17-0.42)	<0.0001
White-Native America	1.55(0.99-2.43)	0.0562	1.56(0.99-2.47)	0.0577	1.76(1.11-2.79)	0.0157
White-Other	0.48(0.34-0.67)	<0.0001	0.47(0.34-0.66)	<0.0001	0.28(0.18-0.44)	<0.0001
Primary expected pay						
Medicare-Medicaid	0.14(0.11-0.18)	<0.0001	0.45(0.34-0.58)	<0.0001	0.42(0.32-0.56)	<0.0001
Medicare-Private ins	0.15(0.12-0.20)	<0.0001	0.36(0.27-0.47)	<0.0001	0.34(0.25-0.45)	<0.0001
Medicare-Self-pay	0.16(0.11-0.23)	<0.0001	0.55(0.37-0.82)	0.0036	0.56(0.37-0.86)	0.0086
Medicare- No charge	0.39(0.16-0.99)	0.0466	1.16(0.46-2.94)	0.7570	1.24(0.48-3.17)	0.6549
Medicare- Other	0.24(0.17-0.34)	<0.0001	0.66(0.46-0.95)	0.0263	0.69(0.47-1.01)	0.0573
Median household income						
<25-25~49	1.02(0.89-1.17)	0.7725	0.99(0.86-1.14)	0.8522	0.99(0.85-1.15)	0.8837
<25-50~74	0.94(0.82-1.09)	0.4087	0.82(0.71-0.96)	0.0115	0.78(0.67-0.92)	0.0035
<25-≥75	0.74(0.63-0.86)	0.0002	0.56(0.47-0.66)	<0.0001	0.59(0.49-0.71)	<0.0001
Control/ownership of hospital						
Government non-federal- Private not for profit	1.03(0.88-1.20)	0.6928			1.06(0.89-1.26)	0.5044
Government non-federal- Private investor owned	0.37(0.28-0.48)	<0.0001			0.75(0.56-0.99)	0.0449
Bed size of hospital						
Small-Medium	2.27(1.69-3.05)	<0.0001	2.30(1.71-3.09)	<0.0001	2.32(1.70-3.16)	<0.0001
Small-Large	4.57(3.47-6.02)	<0.0001	4.12(3.13-5.42)	<0.0001	4.09(3.06-5.47)	<0.0001
Teaching hospital	4.18(3.62-4.82)	<0.0001	3.06(2.62-3.57)	<0.0001	2.81(2.39-3.30)	<0.0001
Urban hospital	5.43(3.76-7.86)	<0.0001	2.60(1.75-3.86)	<0.0001	3.65(2.26-5.90)	<0.0001
Region of hospital						
Northeast-Midwest	1.08(0.93-1.27)	0.3054	1.06(0.90-1.25)	0.4630	1.22(1.03-1.45)	0.0246
Northeast-South	0.73(0.63-0.84)	<0.0001	0.83(0.71-0.96)	0.0151	0.89(0.76-1.06)	0.1879
Northeast-West	0.66(0.56-0.78)	<0.0001	0.95(0.80-1.13)	0.5580	1.01(0.84-1.21)	0.9466

Variables	Unadjusted Model		Multivariate Model 1		Multivariate Model 2	
	OR (95% CI)	P value	OR (95% CI)	P value	OR (95% CI)	P value
Gestational hypertension	3.72(3.32-4.18)	<0.0001	2.70(2.35-3.11)	<0.0001	2.84(2.45-3.30)	<0.0001
Pre-eclampsia/eclampsia	2.52(2.13-2.99)	<0.0001	0.67(0.55-0.83)	0.0001	0.74(0.60-0.92)	0.0057
Gestational diabetes	0.96(0.69-1.35)	0.8306	0.52(0.37-0.74)	0.0002	0.60(0.42-0.84)	0.0029
Multiple pregnancy	0.30(0.12-0.73)	0.0083	0.18(0.07-0.45)	0.0002	0.21(0.08-0.51)	0.0006
Acquired immune deficiency syndrome	-	0.9290				
Alcohol abuse	6.91(4.17-11.46)	<0.0001	2.93(1.74-4.94)	<0.0001		
Deficiency anemias	1.44(1.22-1.69)	<0.0001	0.58(0.47-0.72)	<0.0001	0.60(0.48-0.76)	<0.0001
Rheumatoid arthritis/collagen vascular diseases	12.14(9.15-16.09)	<0.0001	3.24(2.41-4.36)	<0.0001	3.77(2.80-5.08)	<0.0001
Chronic blood loss anemia	2.08(1.83-2.37)	<0.0001	1.92(1.62-2.27)	<0.0001	1.72(1.43-2.08)	<0.0001
Chronic pulmonary disease	1.94(1.59-2.36)	<0.0001				
Coagulopathy	3.76(3.03-4.66)	<0.0001	1.80(1.44-2.24)	<0.0001	1.53(1.19-1.96)	0.0009
Depression	4.88(4.13-5.77)	<0.0001	2.31(1.94-2.75)	<0.0001	2.23(1.85-2.69)	<0.0001
Drug abuse	2.46(1.93-3.14)	<0.0001			1.42(1.09-1.84)	0.0089
Hypothyroidism	2.01(1.59-2.54)	<0.0001				
Liver disease	6.75(4.36-10.45)	<0.0001	2.38(1.52-3.73)	0.0001	2.59(1.64-4.08)	<0.0001
Lymphoma	-	0.9436				
Metastatic cancer	-	0.9443				
Other neurological disorders	4.65(3.55-6.09)	<0.0001	1.62(1.21-2.15)	0.0010	1.60(1.19-2.17)	0.0021
Obesity	2.92(2.53-3.37)	<0.0001	1.81(1.56-2.10)	<0.0001	1.67(1.42-1.96)	<0.0001
Paralysis	19.96(12.00-33.20)	<0.0001	4.05(2.33-7.04)	<0.0001	4.06(2.31-7.15)	<0.0001
Psychoses	1.35(0.87-2.10)	0.1817	0.57(0.37-0.90)	0.0154	0.61(0.39-0.96)	0.0334
Solid tumor without metastasis	-	0.9276				
Peptic ulcer disease excluding bleeding	-	0.9554				
Valvular disease	151.53(135.39-169.61)	<0.0001	86.48(76.77-97.43)	<0.0001	81.48(71.65-92.66)	<0.0001
Weight loss	10.91(6.42-18.53)	<0.0001	2.62(1.51-4.56)	0.0006	3.13(1.79-5.46)	<0.0001

Supplementary Table 13. Association of Clinical Factors with Acute Myocardial Infarction During Pregnancy and Puerperium

Variables	Unadjusted Model		Multivariate Model 1		Multivariate Model 2	
	OR (95% CI)	P value	OR (95% CI)	P value	OR (95% CI)	P value
Age group						
18~24-25~29	1.63(1.45-1.83)	<0.0001	1.75(1.56-1.97)	<0.0001	1.81(1.59-2.05)	<0.0001
18~24-30~34	2.91(2.61-3.24)	<0.0001	3.30(2.95-3.68)	<0.0001	3.46(3.07-3.90)	<0.0001
18~24-35~39	5.03(4.50-5.61)	<0.0001	5.40(4.82-6.05)	<0.0001	5.84(5.17-6.61)	<0.0001
18~24-40~44	12.01(10.60-13.61)	<0.0001	11.48(10.09-13.07)	<0.0001	11.56(10.05-13.29)	<0.0001
18~24-≥45	42.78(35.36-51.77)	<0.0001	33.14(27.16-40.43)	<0.0001	35.93(29.10-44.35)	<0.0001
Race						
White-Black	2.35(2.18-2.52)	<0.0001	1.65(1.52-1.79)	<0.0001	1.82(1.67-1.98)	<0.0001
White-Hispanic	0.79(0.72-0.87)	<0.0001	0.81(0.73-0.90)	<0.0001	0.87(0.78-0.96)	0.0087
White- Asian or Pacific Islander	0.87(0.74-1.03)	0.1034	0.93(0.79-1.10)	0.4002	0.99(0.84-1.18)	0.9399
White-Native America	1.31(0.93-1.84)	0.1206	1.32(0.94-1.86)	0.1082	1.50(1.07-2.11)	0.0203
White-Other	1.16(1.00-1.35)	0.0537	1.12(0.96-1.31)	0.1421	1.19(1.01-1.39)	0.0386
Primary expected pay						
Medicare-Medicaid	0.20(0.17-0.24)	<0.0001	0.64(0.54-0.76)	<0.0001	0.69(0.57-0.83)	<0.0001
Medicare-Private ins	0.17(0.15-0.20)	<0.0001	0.51(0.43-0.60)	<0.0001	0.54(0.45-0.66)	<0.0001
Medicare-Self-pay	0.24(0.19-0.30)	<0.0001	0.74(0.59-0.93)	0.0110	0.81(0.63-1.05)	0.1081
Medicare- No charge	0.54(0.32-0.91)	0.0200	1.29(0.76-2.18)	0.3477	1.52(0.89-2.59)	0.1258
Medicare- Other	0.25(0.20-0.31)	<0.0001	0.85(0.67-1.07)	0.1636	0.97(0.75-1.24)	0.7907
Median household income						
<25-25~49	0.82(0.75-0.88)	<0.0001	0.93(0.86-1.01)	0.1027	0.93(0.85-1.02)	0.1081
<25-50~74	0.69(0.63-0.75)	<0.0001	0.75(0.68-0.81)	<0.0001	0.73(0.67-0.81)	<0.0001
<25-≥75	0.63(0.57-0.68)	<0.0001	0.62(0.56-0.69)	<0.0001	0.64(0.57-0.71)	<0.0001
Control/ownership of hospital						
Government non-federal- Private not for profit	0.91(0.83-0.99)	0.0320				
Government non-federal- Private investor owned	0.80(0.71-0.90)	0.0002				
Bed size of hospital						
Small-Medium	1.49(1.31-1.70)	<0.0001	1.39(1.22-1.59)	<0.0001	1.25(1.09-1.42)	0.0012
Small-Large	2.16(1.91-2.43)	<0.0001	1.88(1.66-2.12)	<0.0001	1.65(1.46-1.87)	<0.0001
Teaching hospital	1.72(1.61-1.83)	<0.0001	1.12(1.04-1.20)	0.0031		
Urban hospital	2.47(2.13-2.87)	<0.0001	1.92(1.63-2.25)	<0.0001	1.68(1.44-1.97)	<0.0001
Region of hospital						
Northeast-Midwest	1.16(1.05-1.29)	0.0036	1.35(1.22-1.50)	<0.0001	1.22(1.09-1.37)	0.0005
Northeast-South	1.22(1.11-1.34)	<0.0001	1.32(1.20-1.45)	<0.0001	1.26(1.15-1.39)	<0.0001

Variables	Unadjusted Model		Multivariate Model 1		Multivariate Model 2	
	OR (95% CI)	P value	OR (95% CI)	P value	OR (95% CI)	P value
Northeast-West	0.77(0.69-0.86)	<0.0001	0.96(0.86-1.07)	0.4723	0.95(0.84-1.06)	0.3395
Gestational hypertension	3.04(2.83-3.26)	<0.0001	1.75(1.63-1.89)	<0.0001	1.79(1.65-1.93)	<0.0001
Pre-eclampsia/eclampsia	2.68(2.43-2.95)	<0.0001				
Gestational diabetes	2.19(1.91-2.50)	<0.0001				
Multiple pregnancy	1.08(0.82-1.43)	0.5830	0.65(0.49-0.86)	0.0027	0.72(0.55-0.96)	0.0242
Acquired immune deficiency syndrome	9.20(4.94-17.15)	<0.0001	2.12(1.13-3.97)	0.0192	2.62(1.40-4.92)	0.0027
Alcohol abuse	8.70(6.68-11.33)	<0.0001	2.20(1.67-2.91)	<0.0001	1.94(1.42-2.64)	<0.0001
Deficiency anemias	2.38(2.20-2.58)	<0.0001	1.19(1.06-1.34)	0.0036	1.17(1.04-1.33)	0.0111
Rheumatoid arthritis/collagen vascular diseases	3.68(2.75-4.93)	<0.0001				
Chronic blood loss anemia	2.53(2.36-2.72)	<0.0001	1.49(1.35-1.66)	<0.0001	1.56(1.39-1.74)	<0.0001
Chronic pulmonary disease	2.49(2.25-2.76)	<0.0001	1.37(1.23-1.53)	<0.0001	1.26(1.12-1.41)	0.0001
Coagulopathy	7.34(6.67-8.07)	<0.0001	4.37(3.96-4.82)	<0.0001	4.45(4.01-4.94)	<0.0001
Depression	2.43(2.13-2.77)	<0.0001	1.25(1.09-1.43)	0.0012	1.40(1.22-1.61)	<0.0001
Drug abuse	4.19(3.74-4.69)	<0.0001	2.51(2.22-2.84)	<0.0001	2.66(2.34-3.03)	<0.0001
Hypothyroidism	2.15(1.88-2.46)	<0.0001	1.35(1.18-1.55)	<0.0001	1.38(1.19-1.59)	<0.0001
Liver disease	5.56(4.20-7.36)	<0.0001	1.54(1.15-2.06)	0.0035	1.60(1.18-2.17)	0.0024
Lymphoma	-	0.9036				
Metastatic cancer	48.46(29.34-80.02)	<0.0001	11.83(7.07-19.78)	<0.0001	14.57(8.70-24.38)	<0.0001
Other neurological disorders	7.20(6.33-8.20)	<0.0001	3.00(2.62-3.45)	<0.0001	3.23(2.80-3.73)	<0.0001
Obesity	3.41(3.15-3.69)	<0.0001	2.01(1.85-2.19)	<0.0001	2.01(1.84-2.20)	<0.0001
Paralysis	25.15(19.28-32.82)	<0.0001	4.51(3.35-6.06)	<0.0001	2.48(1.68-3.67)	<0.0001
Psychoses	3.04(2.55-3.62)	<0.0001				
Solid tumor without metastasis	-	0.8765				
Peptic ulcer disease excluding bleeding	24.58(10.24-59.00)	<0.0001	5.99(2.45-14.67)	<0.0001	6.78(2.77-16.61)	<0.0001
Valvular disease	33.63(30.29-37.35)	<0.0001	16.82(15.08-18.76)	<0.0001	17.34(15.46-19.45)	<0.0001
Weight loss	33.93(28.35-40.61)	<0.0001	8.62(7.07-10.50)	<0.0001	8.47(6.85-10.47)	<0.0001

Supplementary Table 14. Association of Clinical Factors with Pulmonary Embolism During Pregnancy and Puerperium

Variables	Unadjusted Model		Multivariate Model 1		Multivariate Model 2	
	OR (95% CI)	P value	OR (95% CI)	P value	OR (95% CI)	P value
Age group						
18~24-25~29	1.11(1.08-1.15)	<0.0001	1.20(1.16-1.23)	<0.0001	1.29(1.20-1.38)	<0.0001
18~24-30~34	1.12(1.08-1.15)	<0.0001	1.26(1.23-1.30)	<0.0001	1.33(1.24-1.44)	<0.0001
18~24-35~39	1.49(1.44-1.54)	<0.0001	1.62(1.57-1.68)	<0.0001	1.72(1.58-1.88)	<0.0001
18~24-40~44	1.87(1.77-1.97)	<0.0001	1.90(1.80-2.01)	<0.0001	2.26(1.99-2.57)	<0.0001
18~24-≥45	2.70(2.32-3.15)	<0.0001	2.34(2.00-2.73)	<0.0001	5.51(4.27-7.11)	<0.0001
Race						
White-Black	1.94(1.89-1.99)	<0.0001	1.48(1.44-1.52)	<0.0001	1.39(1.31-1.49)	<0.0001
White-Hispanic	0.64(0.62-0.66)	<0.0001	0.61(0.59-0.63)	<0.0001	0.53(0.48-0.57)	<0.0001
White- Asian or Pacific Islander	0.46(0.43-0.50)	<0.0001	0.49(0.45-0.52)	<0.0001	0.48(0.40-0.57)	<0.0001
White-Native America	0.95(0.83-1.08)	0.4082	0.86(0.75-0.98)	0.0199	0.60(0.42-0.85)	0.0047
White-Other	0.88(0.83-0.93)	<0.0001	0.84(0.80-0.89)	<0.0001	0.69(0.60-0.80)	<0.0001
Primary expected pay						
Medicare-Medicaid	0.42(0.39-0.45)	<0.0001	0.83(0.77-0.90)	<0.0001	1.21(1.00-1.46)	0.0530
Medicare-Private ins	0.32(0.30-0.35)	<0.0001	0.67(0.62-0.73)	<0.0001	0.86(0.71-1.05)	0.1327
Medicare-Self-pay	0.41(0.37-0.45)	<0.0001	0.97(0.88-1.07)	0.5297	1.77(1.41-2.22)	<0.0001
Medicare- No charge	0.53(0.42-0.68)	<0.0001	1.16(0.91-1.48)	0.2380	2.31(1.43-3.73)	0.0006
Medicare- Other	0.38(0.35-0.42)	<0.0001	0.82(0.74-0.90)	<0.0001	1.24(0.98-1.57)	0.0787
Median household income						
<25-25~49	0.91(0.89-0.94)	<0.0001	1.03(1.00-1.06)	0.0744	0.91(0.85-0.97)	0.0063
<25-50~74	0.79(0.77-0.81)	<0.0001	0.90(0.87-0.92)	<0.0001	0.73(0.68-0.78)	<0.0001
<25-≥75	0.72(0.70-0.74)	<0.0001	0.85(0.82-0.88)	<0.0001	0.71(0.65-0.77)	<0.0001
Control/ownership of hospital						
Government non-federal- Private not for profit	0.90(0.87-0.93)	<0.0001	0.89(0.86-0.92)	<0.0001	0.82(0.76-0.88)	<0.0001
Government non-federal- Private investor owned	0.80(0.77-0.83)	<0.0001	0.99(0.95-1.04)	0.7855	0.82(0.74-0.91)	0.0002
Bed size of hospital						
Small-Medium	1.16(1.11-1.20)	<0.0001	1.17(1.13-1.22)	<0.0001	1.05(0.95-1.15)	0.3460
Small-Large	1.33(1.29-1.38)	<0.0001	1.28(1.24-1.33)	<0.0001	1.28(1.17-1.39)	<0.0001
Teaching hospital	1.30(1.27-1.33)	<0.0001			1.27(1.20-1.35)	<0.0001
Urban hospital	1.28(1.23-1.33)	<0.0001	1.22(1.17-1.27)	<0.0001	1.72(1.52-1.95)	<0.0001
Region of hospital						
Northeast-Midwest	1.08(1.04-1.12)	<0.0001	1.06(1.03-1.10)	0.0003	1.17(1.07-1.27)	0.0003
Northeast-South	0.94(0.91-0.97)	<0.0001	0.89(0.87-0.92)	<0.0001	1.04(0.96-1.12)	0.3048
Northeast-West	0.80(0.77-0.83)	<0.0001	0.95(0.91-0.98)	0.0032	1.05(0.96-1.14)	0.3140

Variables	Unadjusted Model		Multivariate Model 1		Multivariate Model 2	
	OR (95% CI)	P value	OR (95% CI)	P value	OR (95% CI)	P value
Gestational hypertension	1.62(1.58-1.67)	<0.0001	1.19(1.15-1.24)	<0.0001	0.52(0.46-0.58)	<0.0001
Pre-eclampsia/eclampsia	1.41(1.35-1.47)	<0.0001	0.77(0.73-0.81)	<0.0001	1.44(1.24-1.69)	<0.0001
Gestational diabetes	1.15(1.09-1.23)	<0.0001	0.80(0.75-0.85)	<0.0001	0.50(0.42-0.60)	<0.0001
Multiple pregnancy	0.98(0.88-1.08)	0.6557	0.71(0.64-0.78)	<0.0001		
Acquired immune deficiency syndrome	5.02(3.76-6.69)	<0.0001	1.58(1.18-2.12)	0.0021		
Alcohol abuse	2.72(2.32-3.19)	<0.0001				
Deficiency anemias	2.90(2.83-2.98)	<0.0001			1.77(1.62-1.94)	<0.0001
Rheumatoid arthritis/collagen vascular diseases	5.25(4.83-5.71)	<0.0001	2.43(2.23-2.66)	<0.0001	2.04(1.65-2.51)	<0.0001
Chronic blood loss anemia	3.54(3.47-3.62)	<0.0001	2.66(2.59-2.72)	<0.0001	1.47(1.35-1.61)	<0.0001
Chronic pulmonary disease	2.89(2.79-2.99)	<0.0001	1.82(1.75-1.88)	<0.0001	1.55(1.42-1.68)	<0.0001
Coagulopathy	7.48(7.24-7.72)	<0.0001	5.23(5.06-5.41)	<0.0001	5.17(4.78-5.60)	<0.0001
Depression	2.92(2.80-3.04)	<0.0001	1.81(1.74-1.89)	<0.0001	1.91(1.74-2.10)	<0.0001
Drug abuse	2.25(2.14-2.37)	<0.0001	1.30(1.23-1.37)	<0.0001	2.02(1.83-2.24)	<0.0001
Hypothyroidism	1.51(1.43-1.59)	<0.0001	1.21(1.15-1.28)	<0.0001		
Liver disease	5.04(4.56-5.58)	<0.0001	2.37(2.13-2.62)	<0.0001	2.59(2.10-3.18)	<0.0001
Lymphoma	11.04(8.64-14.12)	<0.0001	4.91(3.78-6.37)	<0.0001	2.96(1.54-5.71)	0.0012
Metastatic cancer	29.56(23.66-36.95)	<0.0001	10.22(8.05-12.98)	<0.0001	21.64(15.15-30.89)	<0.0001
Other neurological disorders	3.88(3.66-4.12)	<0.0001	1.94(1.82-2.06)	<0.0001	2.34(2.07-2.65)	<0.0001
Obesity	2.83(2.75-2.91)	<0.0001	1.98(1.92-2.04)	<0.0001	2.96(2.77-3.16)	<0.0001
Paralysis	10.54(9.16-12.12)	<0.0001	3.53(3.04-4.09)	<0.0001	3.36(2.48-4.55)	<0.0001
Psychoses	2.47(2.32-2.64)	<0.0001	1.34(1.25-1.44)	<0.0001	1.37(1.18-1.58)	<0.0001
Solid tumor without metastasis	6.68(5.23-8.54)	<0.0001	3.96(3.08-5.09)	<0.0001	9.57(6.62-13.84)	<0.0001
Peptic ulcer disease excluding bleeding	5.69(3.06-10.59)	<0.0001				
Valvular disease	10.95(10.33-11.62)	<0.0001	6.78(6.38-7.21)	<0.0001	13.62(12.27-15.13)	<0.0001
Weight loss	17.51(16.08-19.06)	<0.0001	6.29(5.75-6.89)	<0.0001	8.32(7.06-9.81)	<0.0001

Supplementary Table 15. Temporal Trends of Factors Associated with Acute High-risk Chest Pain Diseases During Pregnancy and Puerperium

Variables	2008	2009	2010	2011	2012	2013	2014	2015	2016	2017	Z Value	P Value
Elderly parturient	14.94	14.95	14.56	15.01	15.1	15.63	15.89	16.39	17.19	17.81	-163.67	<0.0001
Gestational hypertension	9.21	9.65	10.21	10.43	10.48	10.85	11.15	11.06	8.95	10.04	-38.56	<0.0001
Acquired immune deficiency syndrome	0.03	0.03	0.04	0.03	0.02	0.02	0.03	0.02	0.02	0.03	16.55	<0.0001
Alcohol abuse	0.16	0.15	0.19	0.16	0.16	0.17	0.18	0.17	0.13	0.11	15.66	<0.0001
Deficiency anemias	7.59	6.84	8.09	8.00	8.74	8.86	9.71	9.63	7.87	9.52	-153.29	<0.0001
Rheumatoid arthritis/collagen vascular diseases	0.24	0.25	0.29	0.29	0.29	0.32	0.35	0.35	0.34	0.36	-46.30	<0.0001
Chronic blood loss anemia	9.43	9.12	10.32	10.53	11.08	11.31	12.33	13.11	13.68	15.10	-358.73	<0.0001
Chronic pulmonary disease	3.42	3.32	3.96	3.84	4.02	4.15	4.49	4.73	4.91	5.23	-187.30	<0.0001
Coagulopathy	1.02	1.18	1.61	1.69	1.80	1.93	1.97	2.09	2.33	2.52	-211.83	<0.0001
Depression	1.94	1.91	2.32	2.30	2.27	2.44	2.70	2.86	2.89	3.34	-168.80	<0.0001
Drug abuse	1.43	1.51	1.73	1.70	1.94	2.15	2.40	2.50	2.53	2.74	-198.91	<0.0001
Hypothyroidism	1.89	1.90	2.15	2.44	2.67	2.86	3.12	3.31	3.41	3.59	-234.73	<0.0001
Liver disease	0.13	0.14	0.17	0.17	0.20	0.20	0.22	0.25	0.33	0.44	-110.35	<0.0001
Lymphoma	0.02	0.02	0.02	0.02	0.02	0.02	0.02	0.02	0.01	0.01	8.77	<0.0001
Metastatic cancer	0.01	0.01	< .01	0.01	0.01	0.01	0.01	0.01	0.01	0.01	-6.99	<0.0001
Other neurological disorders	0.57	0.62	0.65	0.64	0.67	0.72	0.75	0.89	1.46	1.93	-232.30	<0.0001
Obesity	3.36	3.25	4.45	5.16	5.51	6.39	7.25	8.15	9.20	10.70	-616.20	<0.0001
Paralysis	0.05	0.04	0.05	0.05	0.05	0.06	0.05	0.06	0.06	0.07	-17.88	<0.0001
Peripheral vascular disorders	0.02	0.01	0.02	0.02	0.02	0.02	0.03	0.03	0.03	0.03	-30.30	<0.0001
Psychoses	0.86	0.93	1.10	1.07	1.09	1.14	1.23	1.22	1.01	1.08	-42.14	<0.0001
Solid tumor without metastasis	0.03	0.02	0.02	0.03	0.02	0.03	0.03	0.03	0.03	0.03	-10.52	<0.0001

Variables	2008	2009	2010	2011	2012	2013	2014	2015	2016	2017	Z Value	P Value
Peptic ulcer disease excluding bleeding	< .01	< .01	< .01	< .01	< .01	< .01	< .01	0.01	0.02	0.02	-52.36	<0.0001
Valvular disease	0.49	0.40	0.35	0.32	0.28	0.26	0.26	0.25	0.24	0.24	84.29	<0.0001
Weight loss	0.08	0.07	0.10	0.10	0.09	0.10	0.11	0.11	0.08	0.09	-8.41	<0.0001
